# Discovery of Bioactive Indole-Diketopiperazines from the Marine-Derived Fungus *Penicillium brasilianum* Aided by Genomic Information

**DOI:** 10.3390/md17090514

**Published:** 2019-09-01

**Authors:** Ya-Hui Zhang, Ce Geng, Xing-Wang Zhang, Hua-Jie Zhu, Chang-Lun Shao, Fei Cao, Chang-Yun Wang

**Affiliations:** 1 Key Laboratory of Marine Drugs, the Ministry of Education of China, School of Medicine and Pharmacy, Ocean University of China, Qingdao 266003, China; 2 College of Pharmaceutical Sciences, Key Laboratory of Pharmaceutical Quality Control of Hebei Province, Hebei University, Baoding 071002, China; 3 Shandong Provincial Key Laboratory of Synthetic Biology, CAS Key Laboratory of Biofuels at Qingdao Institute of Bioenergy and Bioprocess Technology, Chinese Academy of Sciences, Qingdao 266101, China; 4 Laboratory for Marine Drugs and Bioproducts, Qingdao National Laboratory for Marine Science and Technology, Qingdao 266237, China; 5 Institute of Evolution & Marine Biodiversity, Ocean University of China, Qingdao 266003, China

**Keywords:** biosynthetic gene cluster (BGC), indole-diketopiperazine, *Penicillium brasilianum*, cytotoxicities

## Abstract

Identification and analysis of the whole genome of the marine-derived fungus *Penicillium brasilianum* HBU-136 revealed the presence of an interesting biosynthetic gene cluster (BGC) for non-ribosomal peptide synthetases (NRPS), highly homologous to the BGCs of indole-diketopiperazine derivatives. With the aid of genomic analysis, eight indole-diketopiperazines (**1**−**8**), including three new compounds, spirotryprostatin G (**1**), and cyclotryprostatins F and G (**2** and **3**), were obtained by large-scale cultivation of the fungal strain HBU-136 using rice medium with 1.0% MgCl_2_. The absolute configurations of **1**−**3** were determined by comparison of their experimental electronic circular dichroism (ECD) with calculated ECD spectra. Selective cytotoxicities were observed for compounds **1** and **4** against HL-60 cell line with the IC_50_ values of 6.0 and 7.9 μM, respectively, whereas **2**, **3**, and **5** against MCF-7 cell line with the IC_50_ values of 7.6, 10.8, and 5.1 μM, respectively.

## 1. Introduction

In the past decades, marine-derived fungi have attracted more and more attention to medicinal chemists because of their ability to produce biologically active compounds with diverse structures for drug discovery [1]. Especially, one fungus species could generate structurally versatile natural products because it consists of a large number of biosynthetic gene clusters (BGCs) associated with its secondary metabolism [2,3]. For example, several bioactive compounds have been generated by the same fungus from different sources, including antifungal diketopiperazine-type alkaloids [4], antibacterial dimeric diketopiperazines [5], cytotoxic prenyl asteltoxin derivatives [6], and anti-virus highly oxygenated cyclopiazonic acid-derived alkaloids [7]. However, it has been proven that most of the genes encoding secondary metabolites in fungi are cryptic under certain culture conditions [8]. In recent years, due to the advances in genome sequencing and bioinformatics-based predictions of encoded natural products in BGCs, it has been enabled to obtain structurally-unique secondary metabolites from fungi with the assistance of genomic information [8,9,10].

During our investigation focused on discovering bioactive natural products from marine-derived fungi [11,12,13,14], two known cytotoxic diketopiperazine alkaloids, spirocyclic diketopiperazine alkaloid (**4**) [15] and cyclotryprostatin B (**5**) [16], were discovered from the marine-derived fungus *Penicillium brasilianum* HBU-136 by using rice medium in the first large-scaled fermentation. After gaining these two known diketopiperazine alkaloid derivatives, we tried to locate their BGC in the genome of the fungus HBU-136. A search of the genome sequence using antiSMASH 4.2 found that the strain harbors multiple BGCs for non-ribosomal peptide synthetases (NRPS), one of which shows high similarity to the BGC of fumitremorgins A‒C in the fungus *Aspergillus fumigatus* [17,18,19]. By gene-to-gene comparisons, it is disclosed that the main difference between the BGC in HBU-136 (*ctp*) against in *Aspergillus fumigatus* (*ftm*) lies at the tailoring genes (Table S2 and Figure S45), which suggested that the BGC in HBU-136 may having the ability to produce new derivatives. Based on this information, we tried more fermentation conditions with LC-MS analysis, and finally found three new indole-diketopiperazines (**1**−**3**) from the rice medium with 1.0% MgCl_2_ fermentation. Herein, we report the isolation, structural characterization, putative biosynthetic relation and biological activities of these compounds.

## 2. Results and Discussion

### 2.1. Identification and Analysis of the Indole-Diketopiperazine BGC

The whole genome sequence of *Penicillium brasilianum* HBU-136 was sequenced within HiSeq X10 platform (Illumina, CA, USA) (150 fold) and further assembled into 937 contigs (35.5 Mb) with *de novo* assembly software ABySS and SPAdes. For further accurate BGCs mining and comparison, all the genes were predicted by gene finding software GeneMarks ES/ET and Augustus with self-training. Then, 50 secondary metabolite gene clusters were proposed by using antiSMASH 4.2 as a gene cluster mining tool (Table S1). Thereinto, 12 out of the 50 BGCs were described as NRPS, including a BGC highly homologous to the fumitremorgin BGC (8 out of 9 genes show similarity Table S2) [17,18,19]. This highly similar gene cluster involved an NRPS gene (*ctpNRPS*), three cytochrome P450 genes (*ctpP450-1/2/3*), one oxmethyltransferase gene (*ctpOMT*), two prenyltransferase genes (*ctpPT-1/2*), and one putative oxidase gene (*ctpOx*) (Table S2).

**Figure 1:**
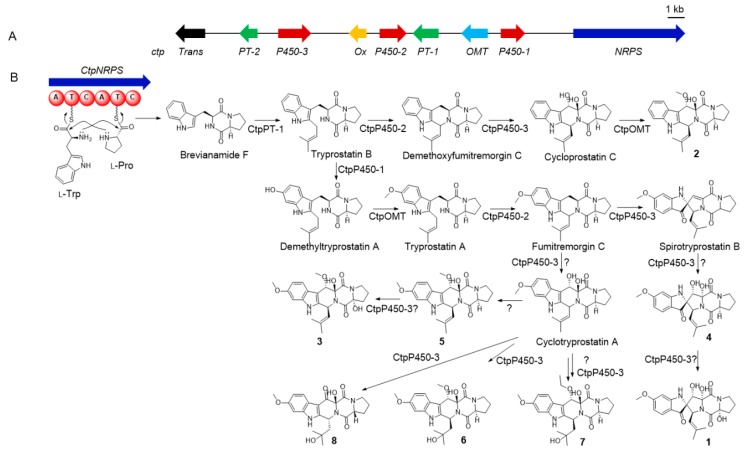
(**A**) The biosynthetic gene cluster of cyclotryprostatin from *Penicillium brasilianum* (*ctp*). (**B**) Proposed biosynthetic pathway of the isolated indole-diketopiperazines.

By gene-to-gene comparison, it suggested that the proposed gene clusters were consistent well with the assembly of the dipeptide skeleton for indole-diketopiperazines (Figure 1). The key biosynthetic step of dipeptide skeleton started from two successive amidation reactions catalyzed by the *ctpNRPS* gene and led to the formation of brevianamide F [20,21]. Then *ctpPT-1* gene mediated the isopentane addition and delivered the product directly to *ctpP450-2* gene, who catalyzed the tetrahydropyridine ring formation, or to *ctpOMT* gene to get the methoxyl first before the ring-formation step [20,21]. The formed cyclotryprostatin skeleton could be further transformed into spirotryprostatin-type by *ctpP450-3* gene via a radical formation and migration eliciting semipinacol-type rearrangement mechanism [22]. P450s, the most versatile biocatalysts in nature, have been proved to catalyze such uncommon reactions during skeleton constructing processes in many cases [23]. After the core skeletons constructed, the structural diversity of both spirotryprostatins and cyclotryprostatins mainly come from the tailoring reactions of *ctpP450-3* gene to complete hydroxylation reactions, *ctpP450-1* gene to catalyze the benzene hydroxylation, and *ctpOMT* gene to mediate the methylation reaction.

**Figure 2:**
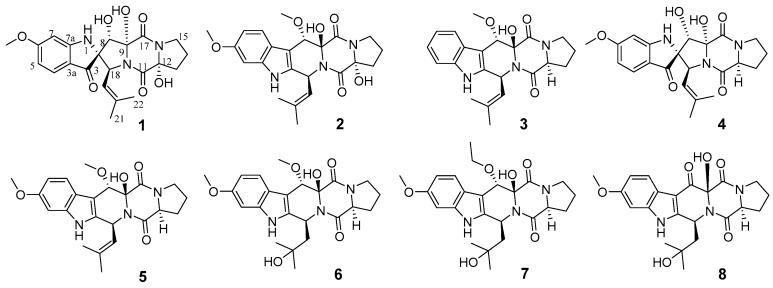
Chemical structures of compounds **1**–**8**.

More intriguingly, the gene *ct*pP*450-3*, whose corresponding gene in *ftm* is the multi-functional gene *ftmG*, was also inferred to encode a muti-functional P450 according to their sequence identity, suggesting that new polyhydroxylated indole-diketopiperazines could be generated by the fungal strain HBU-136. At this point, we tried eight different media conditions (C1–C8) with LC-MS screening to mine more biosynthetic products for *ctp*. Guided by HPLC-MS, it was found that three previously unrecognized compounds exhibiting the typical indole UV spectrum were produced by the strain HBU-136 when 1.0% MgCl_2_ was added to rice medium (C-8). According to previous literature, the addition of MgCl_2_ into the medium was reported to impact the secondary metabolism [13,24], which was confirmed once again by our research. Finally, eight indole-diketopiperazines (**1**–**8**), including three new compounds, spirotryprostatin L (**1**) and cyclotryprostatins F–G (**2**–**3**), and five known analogs, spirocyclic diketopiperazine alkaloid (**4**) [15], cyclotryprostatin B (**5**) [16], 20-hydroxycyclotryprostatin B (**6**) [25], 12*β*-hydroxy-13*α*-ethoxyverruculogen TR-2 (**7**) [26], and an unnamed compound (**8**) [27], were then obtained (Figure 2).

### 2.2. Structure Elucidation

**Figure 3:**
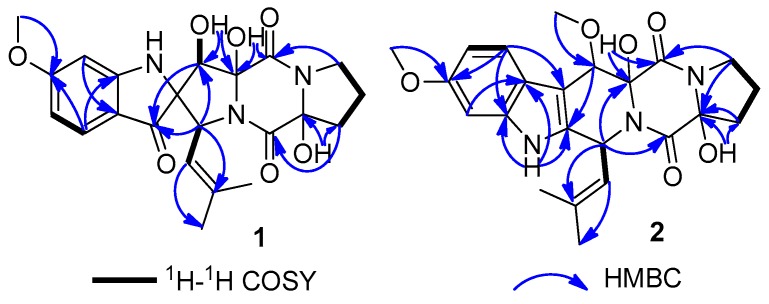
^1^H-^1^H COSY and key HMBC correlations of **1** and **2**. In order to assign the relative configuration of **1**, its NOESY experiment was carried out.

Spirotryprostatin L (**1**), a yellow amorphous powder, was obtained with the molecular formula C_22_H_25_N_3_O_7_ (12 degrees of unsaturation) by positive HRESIMS. The IR absorption bands at 3447 cm^−1^ and 1647 cm^−1^ indicated the presences of hydroxyl and carbonyl groups. The characteristic UV absorption maxima at 220, 249, 287, 387 nm suggested a spirotryprostatin skeleton for **1** [15,16]. The NMR signals in **1** (Table 1 and Table 2) were representative of one keto carbonyl (C-3), two amide carbonyls (C-11 and C-17), one 1,3,4-trisubstituted aromatic unit (C-3a, C-4, C-5, C-6, C-7, and C-7a), and three methyls (one oxygenated) (6-OCH_3_, C-22, and C-21). These structural features suggested a prenylated indole-diketopiperazine nucleus of **1**, structurally similar to the known compound **4** which was previously obtained from the fungus *Aspergillus fumigatus* [15]. A detailed analysis for HMBC correlations of **1** (from 12-OH to C-12 and C-13) (Figure 3) indicated that an additional hydroxyl group [*δ*_H_ 5.14 (1H, brs, 12-OH) and *δ*_C_ 90.2 (C-12)] was attached to C-12. Based on the systematic 2D NMR data analyses, the plane structure of **1** was determined.

**Figure 4:**
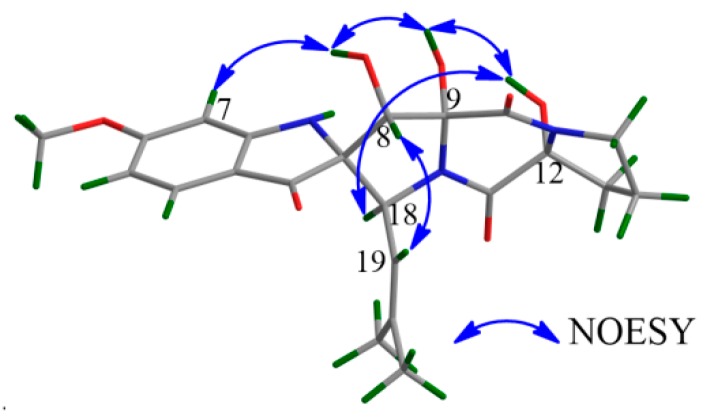
The key NOESY correlations of **1**.

**Figure 5:**
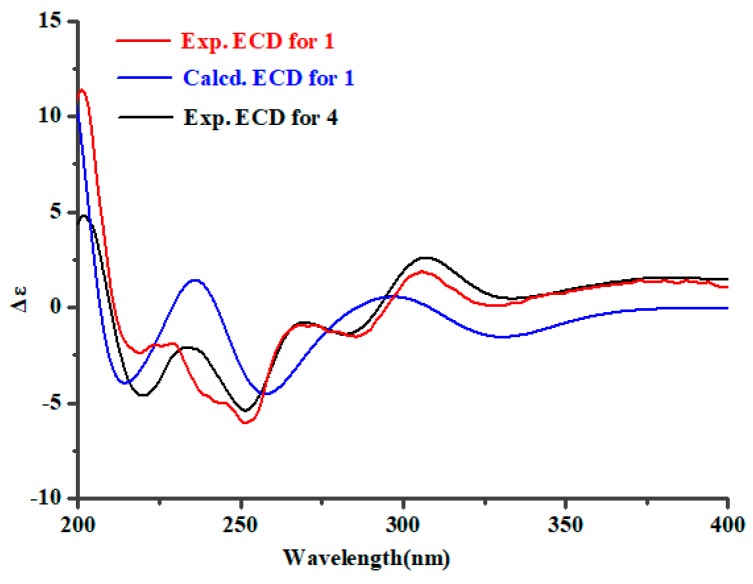
Experimental ECD of **1** and **4**, and calculated ECD of (2*S*,8*S*,9*R*,12*R*,18*S*)-**1**.

The NOESY correlations of **1** between H-7 and 8-OH, 8-OH and 9-OH, 9-OH and 12-OH, 12-OH and H-18, and H-8 and H-19 (Figure 4) suggested that H-18, 8-OH, 9-OH, and 12-OH were located on the same face of the molecule, and H-7 had a *cis* relationship with 8-OH/9-OH/12-OH/H-18. The electronic circular dichroism (ECD) spectrum of **1** was tested and calculated to investigate the absolute configuration of **1**. Time-dependent density functional theory (TD-DFT) method was used for ECD calculation of the molecule (2*S*,8*S*,9*R*,12*R*,18*S*)-**1** at the B3LYP/6-311++G(2d,p)//B3LYP/6-311+G(d) level. Boltzmann statistics with a standard deviation of σ 0.3 eV was used for the ECD simulation. The calculated ECD spectrum of (2*S*,8*S*,9*R*,12*R*,18*S*)-**1** was in good accordance with the experimental ECD data of **1** (Figure 5), revealing that the absolute configuration of **1** should be 2*S*,8*S*,9*R*,12*R*,18*S*. Additionally, the above conclusion was verified by the fact that the experimental ECD spectrum and the specific optical rotation (OR) value of **1** were similar to those of **4** ([*α*]_D_^20^ = + 144.6 (*c* 1.00, MeOH) for **1** vs. [*α*]_D_^20^ = + 147.2 (*c* 0.1, CHCl_3_) for **4**) [15]. Thus, compound **1** was defined as a 12-hydroxylation derivative of **4**.

**Figure 6:**
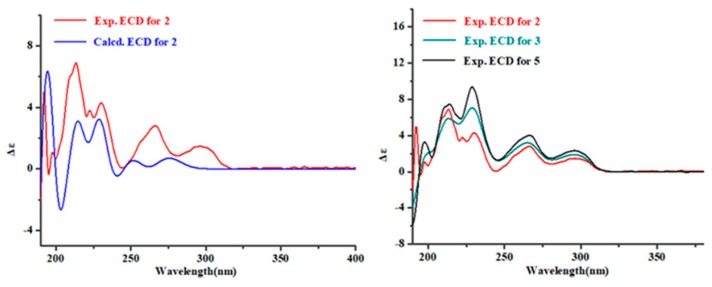
Experimental ECD of **2**, **3** and **5**, and calculated ECD of (8*S*,9*S*,12*R*,18*S*)-**2**.

Cyclotryprostatin F (**2**) was also obtained as a yellow amorphous powder, whose molecular formula was deduced as C_23_H_27_N_3_O_6_ from HRESIMS. The NMR data of **2** (Table 1; Table 2) suggested that it also possessed a diketopiperazine skeleton, which was considered similar to the known compound **5** [16] through detailed NMR analyses of them. Combining the upfield shifted C-11 (*δ*_C_ 166.3 in **2**; *δ*_C_ 167.0 in **5**) and C-14 (*δ*_C_ 19.2 in **2**; *δ*_C_ 22.1 in **5**), and downfield shifted C-12 (*δ*_C_ 86.7 in **2**; *δ*_C_ 59.9 in **5**) and C-13 (*δ*_C_ 36.5 in **2**; *δ*_C_ 29.7 in **5**) of **2** with the change of the molecular weight from **5** to **2** suggested that **2** was a 12-hydroxylation derivative of **5**. This deduction could be confirmed by the key HMBC correlations from H-13 and H-15 to C-12 in **2** (Figure 3). Similar ^1^H-^1^H coupling constants, NOESY correlations and ECD Cotton effects (Figure 6) of **2** and **5** implied that **2** had the 8*S*,9*S*,12*R*,18*S* absolute configuration, which was also confirmed by TD-DFT ECD calculation of (8*S*,9*S*,12*R*,18*S*)-**2**.

Cyclotryprostatin G (**3**) was also isolated as an analog of **5**, deduced from the similar NMR data of them. Compound **3** was the demethylation derivative of **5** at C-6, which was verified by ^1^H-^1^H COSY (H-4/H-5/H-6/H-7) and HMBC (H-6 and C-4, and H-6 and C-7a) experiments for **3**. The stereochemistry of **3** was suggested to be 8*S*,9*S*,12*S*,18*S* by comparing the NOESY and ECD spectra of **3** to those of **5** (Figure 6).

It is deduced that, the newborn OH-12 in **1** and **3** may be catalyzed by the multi-functional P450 *ctpP450-3* (Figure 1). And the new formed OMe-8 in **23** may be introduced by the methyltransferase encoded gene *ctpOMT* or other genes outside the reported boundary, or they were formed during our isolation processes.

### 2.3. Biological Activities Screening

It has been reported that many indole-diketopiperazine derivatives displayed a wide range of biological effects, such as cytotoxic [16,28] andantibacterial activities [29]. In the present study, all of the isolated compounds (**1**–**8**) were tested for their cytotoxic and antibacterial activities. Among them, **1** and **4** displayed selective cytotoxicities against HL-60 cell line with the IC_50_ values of 6.0 and 7.9 μM, respectively. Whereas compounds **2**, **3**, and **5** exhibited activities against MCF-7 cell line with the IC_50_ values of 7.6, 10.8, and 5.1 μM, respectively. However, all of the metabolites appeared to be inactive in antibacterial and antifungal assays (MIC > 25 μM).

## 3. Materials and Methods

### 3.1. Instrumentation

Optical rotations were measured on a JASCO P-1020 digital polarimeter (Jasco Corp., Tokyo, Japan). UV spectra were recorded on a Thermo Scientific Multiskan GO microplate spectrophotometer (Thermo Scientific Co., Waltham, MA, USA) in MeOH. ECD spectra were recorded on a JASCO J-815 circular dichroism spectrometer (JASCO Electric Co., Ltd., Tokyo, Japan). IR spectra were determined on a Nicolet-Nexus-470 spectrometer (Thermo Electron Co., Madison, WI, USA) using KBr pellets. 1D and 2D NMR spectroscopic data (TMS as an internal standard) were acquired on Agilent DD2-500 (JEOL, Tokyo, Japan) and Bruker AV-600 NMR spectrometer (Bruker BioSpin GmbH Co., Rheinstetten, Germany). ESIMS and HRESIMS spectra were obtained from a Micromass Q-TOF spectrometer (Waters Corp., Manchester, UK), and a Thermo Scientific LTQ Orbitrap XL spectrometer (Thermo Fisher Scientific, Bremen, Germany). Semipreparative HPLC was performed on a Shimadzu LC-20 AT system using a RP-C18 (Waters, 5 μm, 10 × 250 mm) column.

### 3.2. Genome Sequencing and Bioinformatics Analysis

The fungus *Penicillium brasilianum* HBU-136 was cultured in PDB medium for 30 h at 25 °C, then high-quality genomic DNA was isolated from cultured cells using Fungal DNA Kit (OMEGA Bio-Tek, E.Z.N.A.^®^, (Norcross, GA, USA). All the purified DNA was used to construct 350 bp DNA library with DNA library preparation kit (Illumina, CA, USA), and was sequenced on HiSeq X10 instrument, employing paired-end 150 base reads (Mega genomics, CN). *De novo* assembling of the draft genome was performed by using the software ABySS 2.15 (Vancouver, NA, Canada) and SPAdes 3.11 (St. Petersburg, EU, Russia), with multiple k-mers specified as “-k 33,55,77,99,117” and coverage fold of 160X PE clean reads. All the assemble scaffold sequences were tested by quality assessment tool QUAST 4.0, the last draft genome sequence was filtered by over 500 bp and was determined via the largest N50 number. Gene functions were proposed by the online BLAST program (http://blast.ncbi.nlm.nih.gov/) and Conserved Domain Database (CDD) at the NCBI server (https://www.ncbi.nlm.nih.gov/). This Whole Genome Shotgun project of the fungus HBU-136 has been deposited at DDBJ/ENA/GenBank under the accession SSWP00000000. The version described in this paper is version SSWP01000000.

### 3.3. Fungal Material

The fungus *Penicillium brasilianum* HBU-136 was collected from the Bohai Sea (Huanghua, Hebei Province, China, June 2016). The strain with the NCBI GenBank accession number MH377073 was deposited in the College of Pharmaceutical Sciences, Hebei University, China.

### 3.4. Fermentation and Purification

The first fermentation was carried out for the fungus HBU-136 using rice medium (80 mL water and 80 g rice in 1 L Erlenmeyer flasks, 40 flasks) at 28 °C for 45 days. After cultivation, the fermented rice substrate was extracted with the mixture of CH_2_Cl_2_/MeOH (1:1, 500 mL for each flask) for five times and EtOAc for two times successively. The two extract solvents were combined as their TLC profiles were almost the same. The combined extract was concentrated to give a residue (30 g), which was further subjected to silica gel column chromatography (CC), eluting with an EtOAc−petroleum ether stepped gradient elution (0%, 20%, 40%, 60%, 80%, 100%), to offer six fractions Fr.1−Fr.6. Fr.5 was separated by twice Sephadex LH-20 CC [petroleum ether−MeOH−CHCl_3_ (v/v, 2:1:1)] to afford four subfractions Fr.5-1−Fr.5-4. Then, Fr.5-3 was fractionated by Sephadex LH-20 CC [MeOH−CHCl_3_ (v/v, 1:1)] and silica gel CC (stepped gradient, EtOAc−petroleum ether), and further purified by semi-preparative HPLC (MeOH−H_2_O, 50:50, 2 mL/min) to afford **4** (3.1 mg) and **5** (6.7 mg).

The second large-scale fermentation (100 flasks) for the fungus HBU-136 was performed for 45 days in a modified rice medium (including 80 mL water, 80 g rice, and 0.8 g MgCl_2_ in 1 L Erlenmeyer flask). The extract was obtained and purified by the same approaches as first fermentation. Compounds **1** (1.8 mg), **4** (5.2 mg), **5** (4.8 mg), **7** (1.5 mg), and **8** (6.2 mg) were given from fraction Fr.5. Compounds **2** (4.0 mg), **3** (3.2 mg), and **6** (4.3 mg) were isolated from fraction Fr.6.

Spirotryprostatin G (**1**): Yellow amorphous powder; ${\left[\alpha \right]}_{D}^{20}$ = + 44.6 (*c* 1.00, MeOH); UV (MeOH) *λ*_max_ (log *ε*) 204 (4.05), 220 (4.02), 249 (3.96), 287 (3.77), 387 (3.17) nm; CD (0.75 mM, MeOH) *λ*_max_ (Δ*ε*) 201 (*+*20.7), 219 (−4.3), 239 (−3.4), 252 (−10.9), 327 (*+*0.16) nm; IR (KBr) *v*_max_ 3447, 2360 and 1647 cm^−1^; ^1^H and ^13^C NMR, see Table 1; Table 2; HRESIMS *m*/*z* 444.1757 [M + H]^+^ (calcd for C_22_H_26_N_3_O_7_, 444.1765 [M + Na]^+^), 466.1578 [M + Na]^+^ (calcd for C_22_H_25_N_3_O_7_Na, 466.1585 [M + Na]^+^).

Cyclotryprostatin F (**2**): Yellow amorphous powder; ${\left[\alpha \right]}_{D}^{20}$ = + 108 (*c* 1.00, MeOH); UV (MeOH) *λ*_max_ (log *ε*) 220 (4.30), 290 (3.70) nm; CD (0.75 mM, MeOH) *λ*_max_ (Δ*ε*) 213 (*+*13.8), 230 (*+*8.6), 266 (*+*5.6), 319 (−0.01) nm; ^1^H and ^13^C NMR, see Table 1; Table 2; HRESIMS *m*/*z* 440.1837 [M − H]^−^ (calcd for C_23_H_26_N_3_O_6_, 440.1827 [M − H]^−^).

Cyclotryprostatin G (**3**): Yellow amorphous powder; ${\left[\alpha \right]}_{D}^{20}$ = + 101 (*c* 0.95, MeOH); UV (MeOH) *λ*_max_ (log *ε*) 223 (4.45), 272 (3.93) nm; CD (1.05 mM, MeOH) *λ*_max_ (Δ*ε*) 195 (−8.1), 208 (*+*11.2), 228 (*+*5.7), 258 (*+*3.2), 298 (*+*0.2) nm; ^1^H and ^13^C NMR, see Table 1; Table 2; HRESIMS *m*/*z* 418.1737 [M + Na]^+^ (calcd for C_22_H_25_N_3_O_4_Na, 418.1737 [M + Na]^+^).

### 3.5. Biological Assay

Cytotoxic Assay. The cytotoxic activity of compounds **1**–**8** were evaluated in vitro by the MTT method [30], with the concentration of 10 μM. Three human tumor cell lines were used, including human promyelocytic leukemia cell line (HL-60), human colorectal cancer cell line (HCT-116), and human breast cancer cell line (MCF-7), with cisplatinum (DDP) as a positive control.

Antibacterial Assay. The antibacterial activity of the isolated compounds was determined using the conventional broth dilution assay [31] with ciprofloxacin (CIP) as a positive control. Sixteen pathogenic bacterial strains were used, including *Bacillus megaterium*, *Bacillus subtilis*, *Escherichia coli*, *Bacillus anthraci*, *Bacillus cereus*, *Bacterium paratyphosum B*, *Enterobacter aerogenes*, *Micrococcus lysodeikticus*, *Micrococcus luteus*, *Proteusbacillm vulgaris*, *Shigella dysenteriae*, *Psmdomonas aeruginosa*, *Staphylococcus aureus*, *Salmonella typhi*, *Vibrio anguillarum*, and *Vibrio parahemolyticus.* The concentration of compounds **1**–**8** was 25 μM.

### 3.6. ECD Spectrum Measurement and Calculation

The tested compounds **1**–**5** were dissolved in chromatographic methanol at the concentration of 0.5 mg/mL and transferred 300 μL to quartz cuvette (1 cm for width) for tested. First tested the ECD spectrum of methanol as the blank control, then tested for compounds **1**–**5**. The wavelength was set at 190–400 nm with the band width of 1 nm, and Nitrogen should be used throughout the experiment at the flow rate of 3 mL/min. The ECD spectrum was calculated by the TDDFT methodology with a larger basis set at the B3LYP/6-311++G(2d,p) level and simulated using SpecDis 1.62 [32] according to Boltzmann distributions.

## 4. Conclusions

In summary, three new indole-diketopiperazine derivatives (**1**–**3**) showed selective cytotoxicities have been obtained from the marine-derived fungus *P. brasilianum* HBU-136 with the aid of genomic analysis. ECD quantum chemistry calculations were carried out to assign the absolute configurations of the new compounds **1**–**3**. The present research indicated that it is a powerful approach to discover new natural products from marine-derived fungi by the combination of chemical and bioinformatics analyses.

## Figures and Tables

**Table 1:** ^1^H NMR Data (*δ*) of **1**–**3** (500 MHz, CDCl_3_, *J* in Hz).

No.	1	2	3
1		7.86, s	7.97, s
4	7.50, d (8.7)	7.48, d (8.4)	7.58, d (7.6)
5	6.45, dd (8.7, 2.0)	6.80, dd (8.4, 2.0)	7.17, dtd (8.0, 7.6, 0.9)
6			7.17, dtd (8.0, 7.6, 0.9)
7	6.27, d (2.0)	6.86, d (2.0)	7.37, d (7.6)
8	4.70, s	5.02, s	4.79, s
12			4.38, dd (10.7, 6.3)
13	2.35, m	2.40, m	2.50, m
	2.27, m	1.98, m	2.01, m
14	2.15, m	2.10, s	2.12, m
	2.00, m	1.98, m	2.01, m
15	3.74, m	3.80, m	3.76, m
	3.54, m	3.70, m	3.71, m
18	4.83, d (9.5)	6.36, d (9.6)	6.68, d (9.7)
19	4.79, d (9.5)	5.33, d (9.6)	5.57, d (9.7)
21	1.56, s	2.04, s	1.80, s
22	1.79, s	1.77, s	2.07, s
8-OH	4.96, brs		
12-OH	5.14, brs		
6-OCH_3_	3.85, s	3.82, s	
9-OH	8.51, brs		
8-OCH_3_		3.30, s	3.38, s

**Table 2:** ^13^C NMR Data (*δ*) of **1**–**3** (125 MHz, CDCl_3_).

No.	1	2	3
2	75.0, C	132.7, C	135.2, C
3	200.6, C	104.8, C	105.7, C
3a	112.4, C	122.5, C	128.5, C
4	127.4, CH	118.9, CH	118.2, CH
5	110.8, CH	110.1, CH	120.7, CH
6	169.7, C	156.6, C	122.3, CH
7	94.9, CH	95.3, CH	111.3, CH
7a	165.4, C	137.1, C	135.9, C
8	73.9, CH	74.9, CH	76.8, CH
9	86.1, C	85.7, C	84.9, C
11	167.4, C	166.3, C	167.2, C
12	90.2, C	86.7, C	60.1, CH
13	35.1, CH_2_	36.5, CH_2_	29.8, CH_2_
14	21.3, CH_2_	19.2, CH_2_	22.3, CH_2_
15	45.3, CH_2_	45.4, CH_2_	46.0, CH_2_
17	166.0, C	165.9, C	166.0, C
18	55.7, CH	49.3, CH	49.2, CH
19	119.4, CH	122.8, CH	123.6, CH
20	142.4, C	138.2, C	138.2, C
21	26.1, CH_3_	18.4, CH_3_	26.2, CH_3_
22	18.8, CH_3_	26.1, CH_3_	18.4, CH_3_
6-OCH_3_	56.0, CH_3_	55.8, OCH_3_	
8-OCH_3_		57.0, OCH_3_	56.8, OCH_3_

## Supplementary Materials

The following are available online at https://www.mdpi.com/1660-3397/17/9/514/s1, NMR and MS data of **1**–**8**; Supplementary tables and figures for BGC analyses; tables for biological assay.
