# Supplementary material for: Discovery of Bioactive Indole-Diketopiperazines from the Marine-Derived Fungus Penicillium brasilianum Aided by Genomic Information
Source: Mar Drugs. 2019 Sep 1;17(9):514. doi: 10.3390/md17090514 (PMC6781160; doi:10.3390/md17090514)
Supplement: Supplementary file 1 [file marinedrugs-17-00514-s001.pdf]

Discovery of Bioactive Indole-Diketopiperazines from the Marine-Derived Fungus *Penicillium brasilianum* Aided by Genomic Information

Ya-Hui Zhang[1,2,†], Ce Geng[3,†], Xing-Wang Zhang[3], Hua-Jie Zhu[2], Chang-Lun Shao[1,4], Fei Cao[2*] and Chang-Yun Wang[1,4,5*]

[1]Key Laboratory of Marine Drugs, the Ministry of Education of China, School of Medicine and Pharmacy, Ocean University of China, Qingdao 266003, P. R. China; 15689932652@163.com(Y.-H.Z.); shaochanglun@163.com(C.-L.S.); changyun@ouc.edu.cn(C.-Y.W.)

[2]College of Pharmaceutical Sciences, Key Laboratory of Pharmaceutical Quality Control of Hebei Province, Hebei University, Baoding 071002, P. R. China ;hjzhu2017@163.com(H.-J.Z); caofei542927001@163.com(F.C.)

[3]Shandong Provincial Key Laboratory of Synthetic Biology, CAS Key Laboratory of Biofuels at Qingdao Institute of Bioenergy and Bioprocess Technology, Chinese Academy of Sciences, Qingdao 266101, P. R. China; gengce@qibebt.ac.cn(C.G.); 735888678@qq.com(X.-W.Z.)

[4]Laboratory for Marine Drugs and Bioproducts, Qingdao National Laboratory for Marine Science and Technology, Qingdao 266237, People's Republic of China

[5]Institute of Evolution & Marine Biodiversity, Ocean University of China, Qingdao 266003, People's Republic of China.

†These authors contributed equally to this work.

*Correspondence : caofei542927001@163.com (F.C.); changyun@ouc.edu.cn(C.-Y.W.)

Supplementary Information

Figure S1. ^{1}H NMR (500 MHz, $CDCl_3$) spectrum of compound **1**.

Figure S2. Partial ^{1}H NMR (500 MHz, $CDCl_3$) spectrum of compound **1**.

Figure S3. Partial ^{1}H NMR (500 MHz, $CDCl_3$) spectrum of compound **1**.

Figure S4. ^{13}C NMR (125 MHz, $CDCl_3$) spectrum of compound **1**.

Figure S5. HSQC ($CDCl_3$) spectrum of compound **1**.

Figure S6. ^{1}H–^{1}H COSY ($CDCl_3$) spectrum of compound **1**.

Figure S7. HMBC ($CDCl_3$) spectrum of compound **1**.

Figure S8. NOESY ($CDCl_3$) spectrum of compound **1**.

Figure S9. HRESIMS spectrum of compound **1**.

Figure S10. ^{1}H NMR (500 MHz, $CDCl_3$) spectrum of compound **2**.

Figure S11. ^{13}C NMR (125 MHz, $CDCl_3$) spectrum of compound **2**.

Figure S12. Partial ^{13}C NMR (125 MHz, $CDCl_3$) spectrum of compound **2**.

Figure S13. HSQC ($CDCl_3$) spectrum of compound **2**.

Figure S14. ^{1}H–^{1}H COSY ($CDCl_3$) spectrum of compound**2**.

Figure S15. HMBC ($CDCl_3$) spectrum of compound **2**.

Figure S16. NOESY ($CDCl_3$) spectrum of compound **2**.

Figure S17. HRESIMS spectrum of compound **2**.

Figure S18. ^{1}H NMR (500 MHz, $CDCl_3$) spectrum of compound **3**

Figure S19. Partial ^{1}H NMR (500 MHz, $CDCl_3$) spectrum of compound **3**.

Figure S20. Partial ^{1}H NMR (500 MHz, $CDCl_3$) spectrum of compound **3**.

Figure S21. ^{13}C NMR (125 MHz, $CDCl_3$) spectrum of compound **3**

Figure S22. Partial ^{13}C NMR (125 MHz, $CDCl_3$) spectrum of compound **3**.

Figure S23. HSQC ($CDCl_3$) spectrum of compound **3**.

Figure S24. ^{1}H–^{1}H COSY ($CDCl_3$) spectrum of compound **3**.

Figure S25. HMBC ($CDCl_3$) spectrum of compound **3**.

Figure S26. NOESY ($CDCl_3$) spectrum of compound **3**.

Figure S27. HRESIMS spectrum of compound **3**.

Figure S28. ^{1}H NMR (600 MHz, $CDCl_3$) spectrum of compound **4**.

Figure S29. ^{13}C NMR (150 MHz, $CDCl_3$) spectrum of compound **4.**

Figure S30. ESIMS spectrum of compound **4**.

Figure S31. ^{1}H NMR (500 MHz, $CDCl_3$) spectrum of compound **5**.

Figure S32. ^{13}C NMR (125 MHz, $CDCl_3$) spectrum of compound **5.**

Figure S33. ESIMS spectrum of compound **5**.

Figure S34. ^{1}H NMR (600 MHz, $CDCl_3$) spectrum of compound **6**.

Figure S35. ^{13}C NMR (150 MHz, $CDCl_3$) spectrum of compound **6.**

Figure S36. ESIMS spectrum of compound **6**.

Figure S37. ^{1}H NMR (500 MHz, $CDCl_3$) spectrum of compound **7**.

Figure S38. ^{13}C NMR (125 MHz, $CDCl_3$) spectrum of compound **7.**

Figure S39. ESIMS spectrum of compound **7**.

Figure S40. ^{1}H NMR (600 MHz, DMSO-d_6) spectrum of compound **8**.

Figure S41. ^{13}C NMR (150 MHz, DMSO-d_6) spectrum of compound **8.**

Figure S42. ESIMS spectrum of compound **8**.

Figure S43. UV spectra of compounds **1**–**3**.

Figure S44. HPLC at 254 nm of the fermentation extracts from cultures in different media.

Figure S45. Comparison of our compounds BGC with fumitremorgin BGC

Table S1. Proposed NRPS biosynthetic gene clusters (NRPS-BGCs) predicted by fungiSMASH.

Table S2. Proposed functions of genes in *ctp* gene clusters.

Table S3. The coordinate for the lowest-energy conformer [(2*S*,8*S*,9*R*,12*R*,18*S*)-**1**] in ECD calculation.

Table S4. The coordinate for the lowest-energy conformer [(8*S*,9*S*,12*R*,18*S*)-**2**] in ECD calculation.

Table S5. Antibacterial activities data of compounds **1**–**8**.

Table S6. Cytotoxic activity data of compounds **1**–**8**.

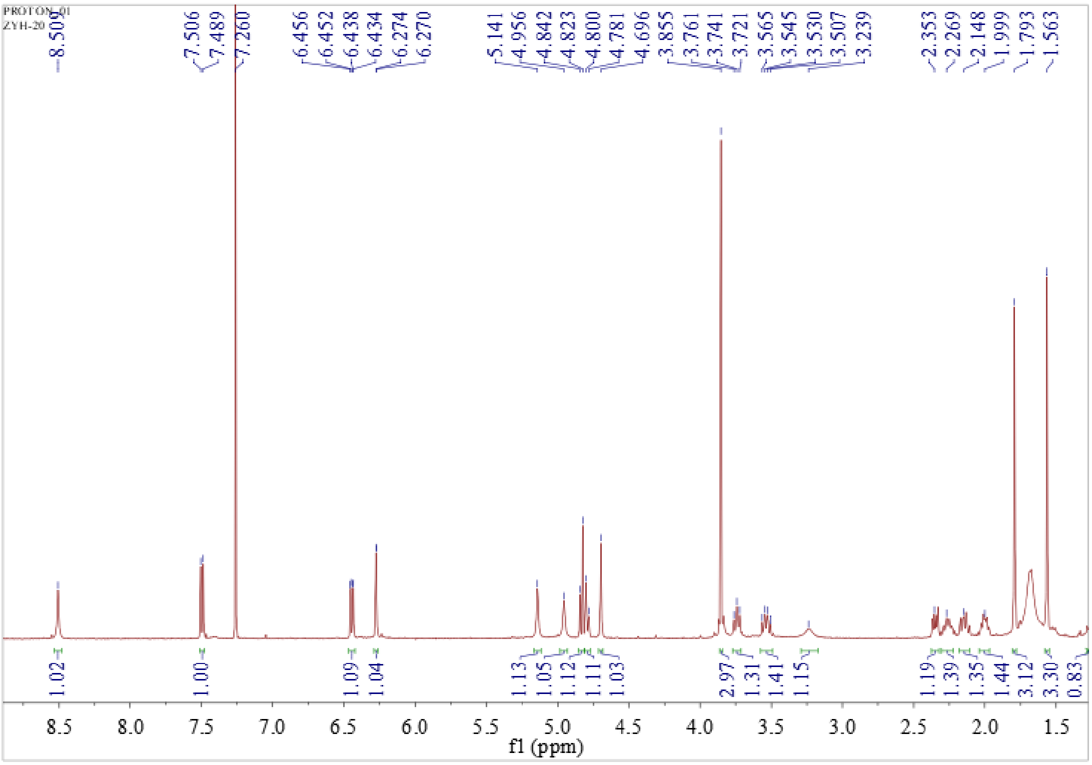

Figure S1. ^{1}H NMR (500 MHz, $CDCl_3$) spectrum of compound **1**.

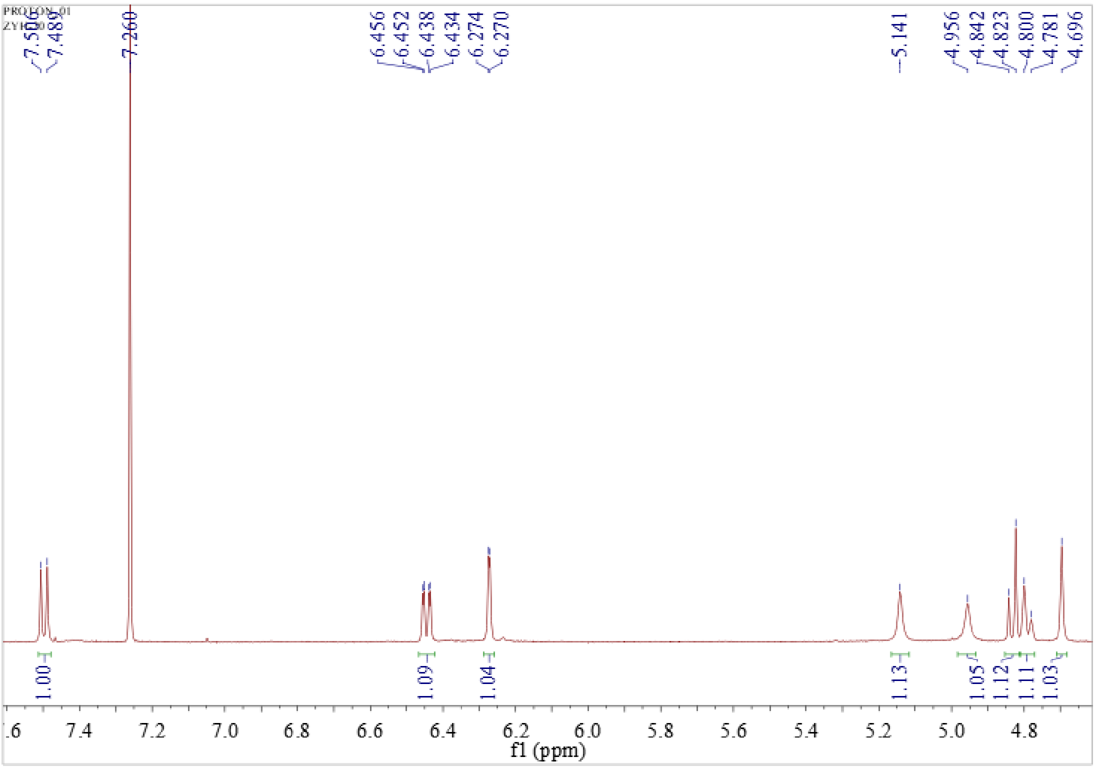

Figure S2. Partial ^{1}H NMR (500 MHz, $CDCl_3$) spectrum of compound **1**.

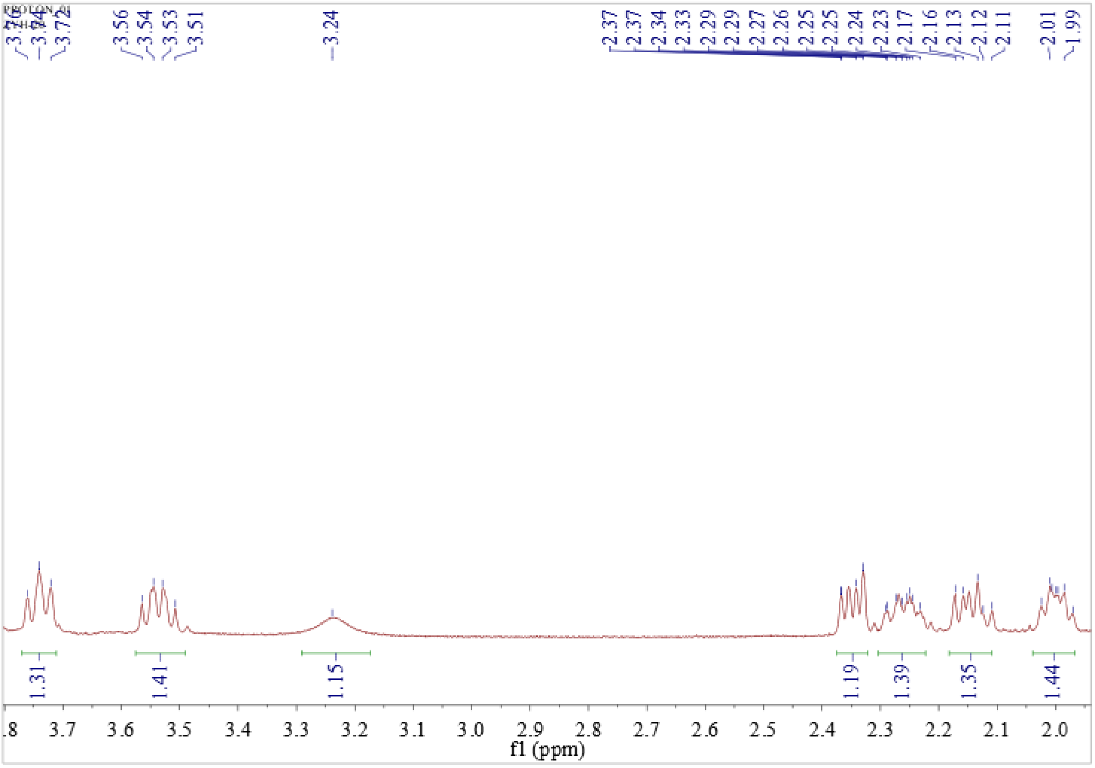

Figure S3. Partial ^{1}H NMR (500 MHz, $CDCl_3$) spectrum of compound **1**

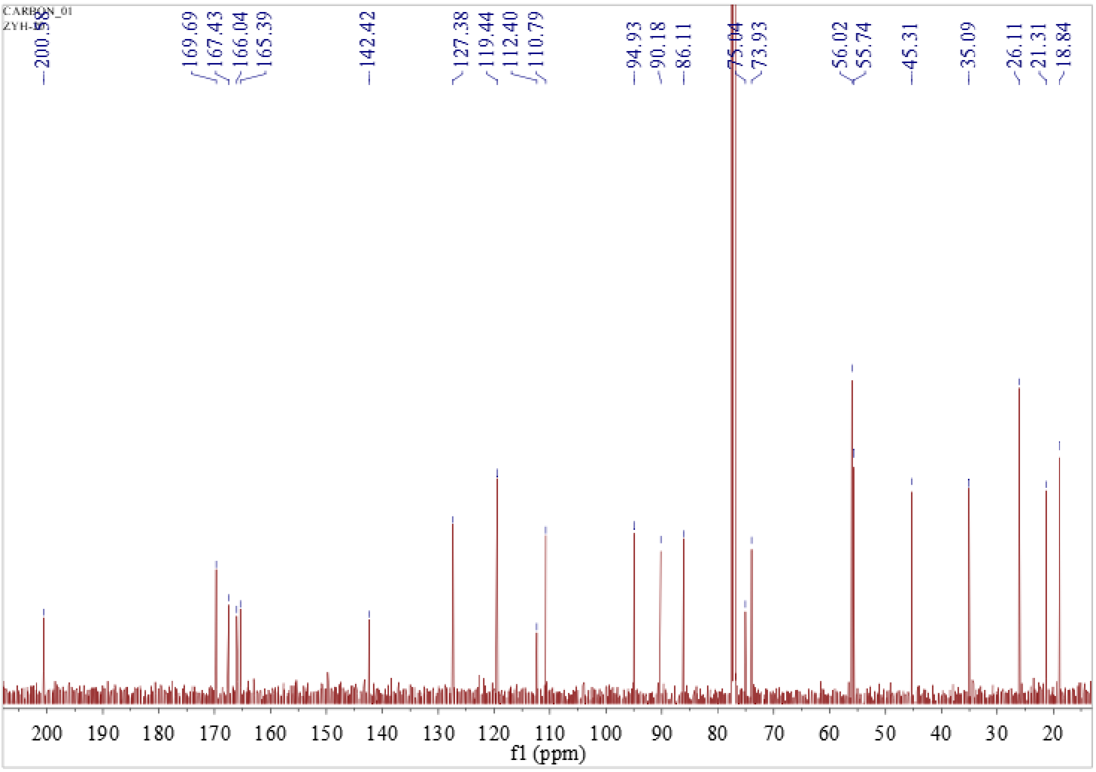

Figure S4. ^{13}C NMR (125 MHz, $CDCl_3$) spectrum of compound **1**.

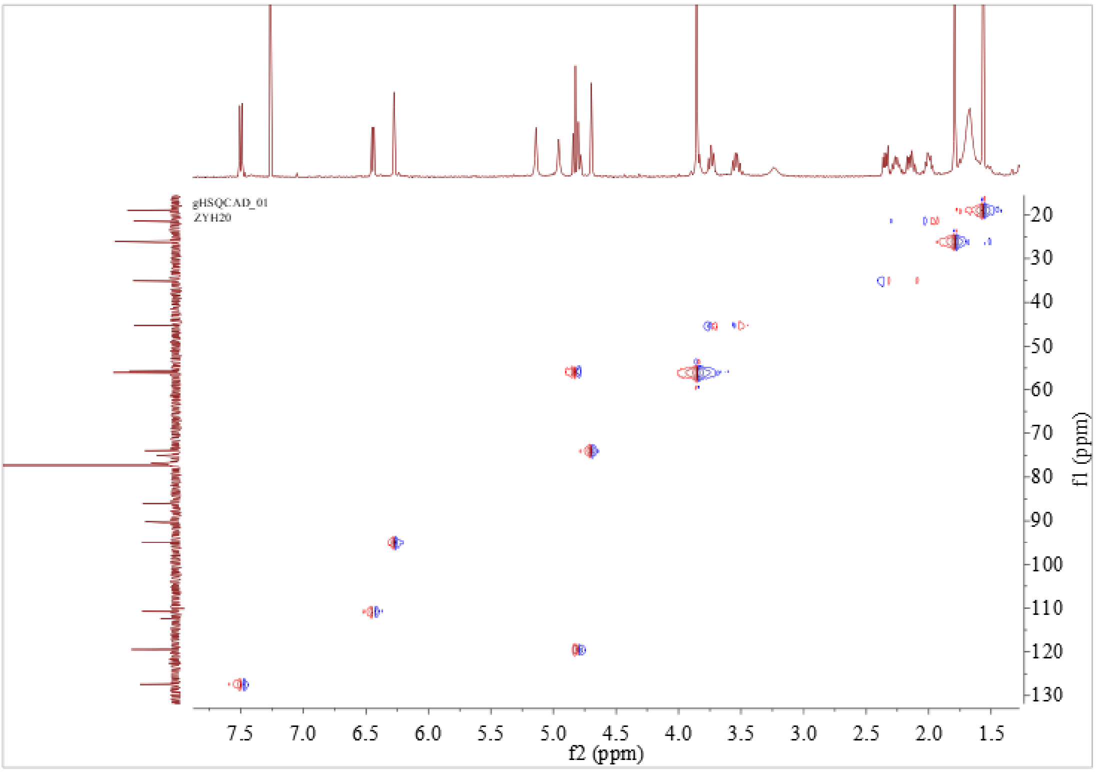

Figure S5. HSQC ($CDCl_3$) spectrum of compound **1**.

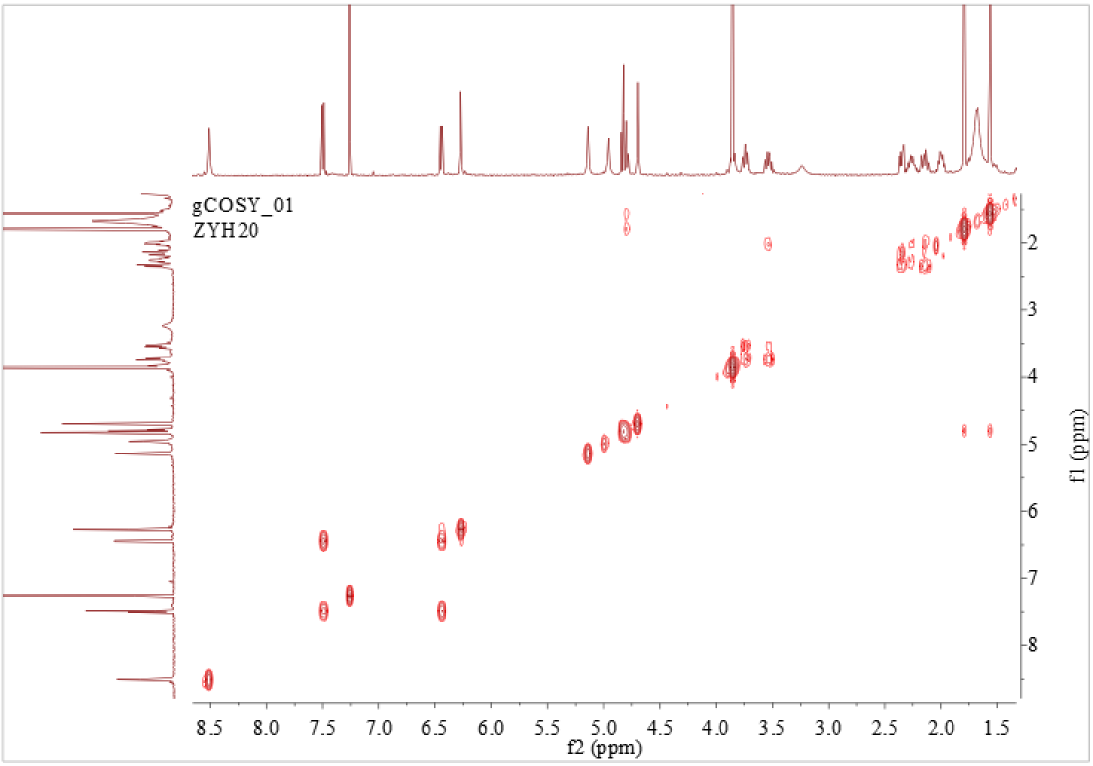

Figure S6. COSY ($CDCl_3$) spectrum of compound **1**.

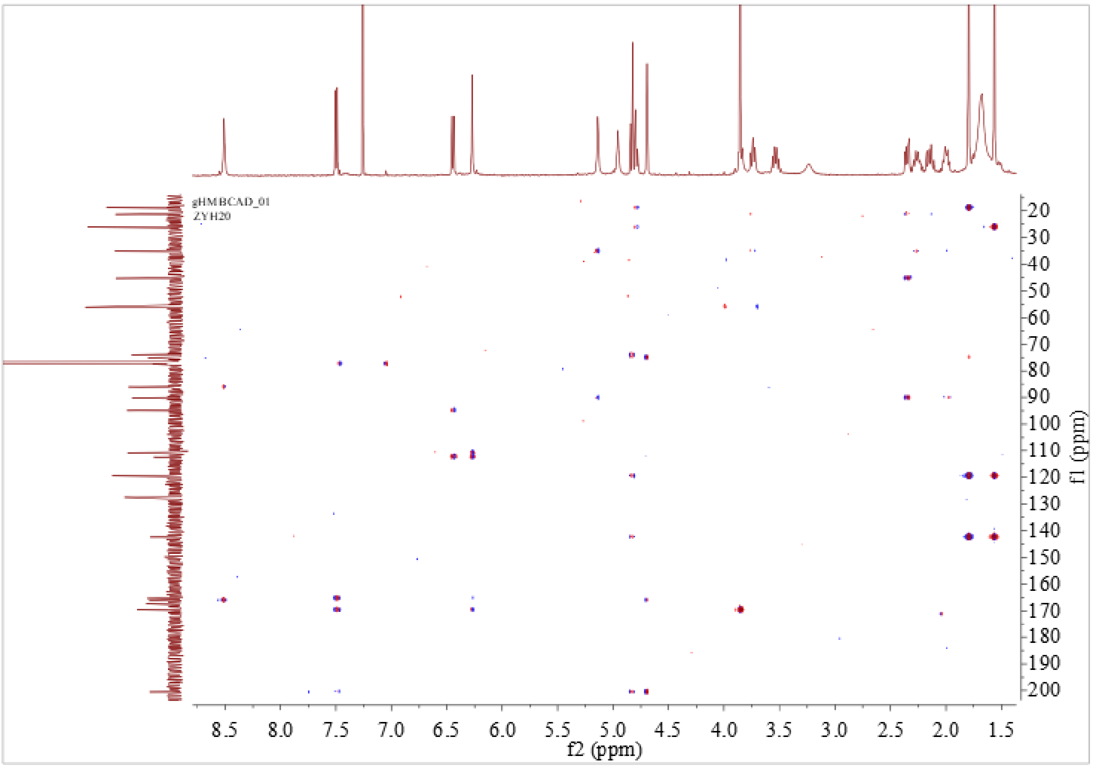

Figure S7. HMBC ($CDCl_3$) spectrum of compound **1**.

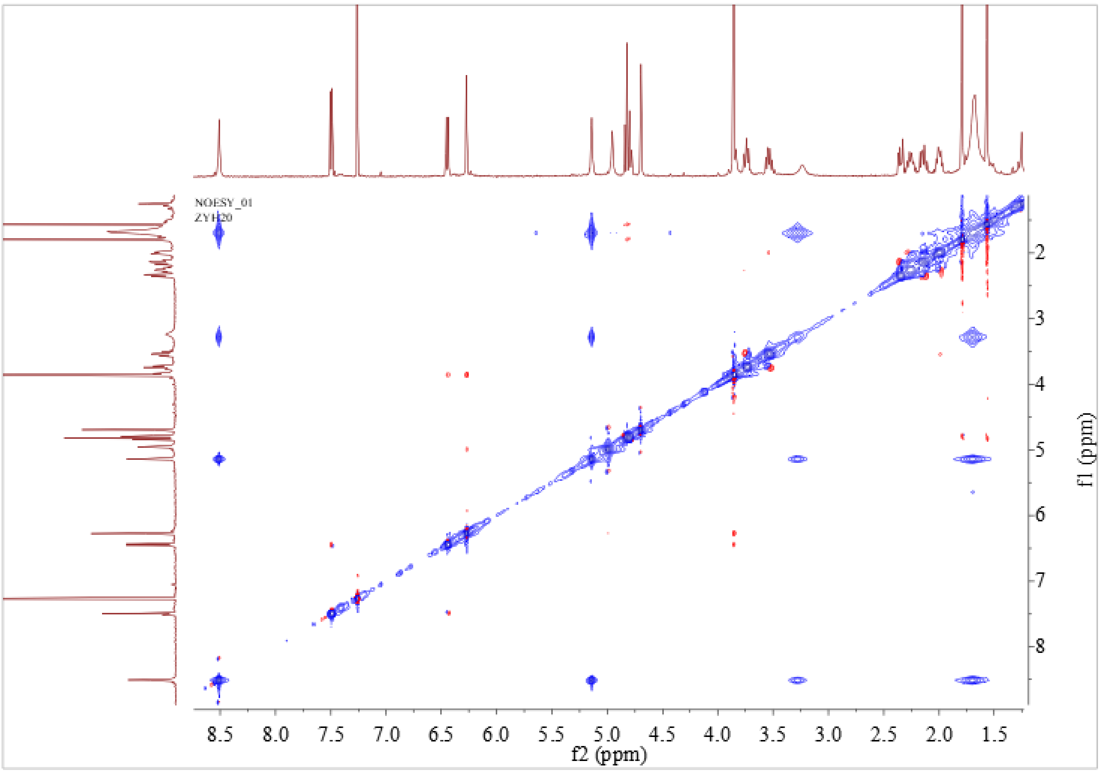

Figure S8. NOESY ($CDCl_3$) spectrum of compound **1**.

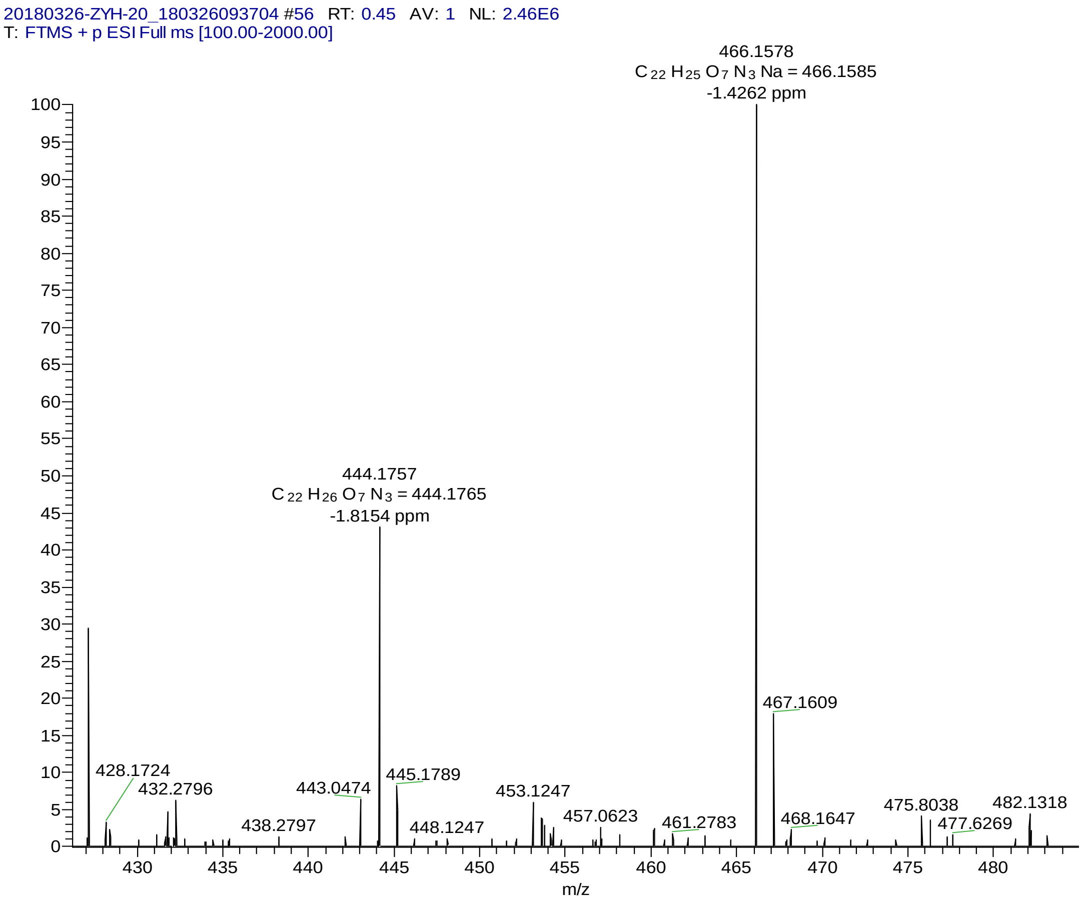

Figure S9. HRESIMS spectrum of compound **1**.

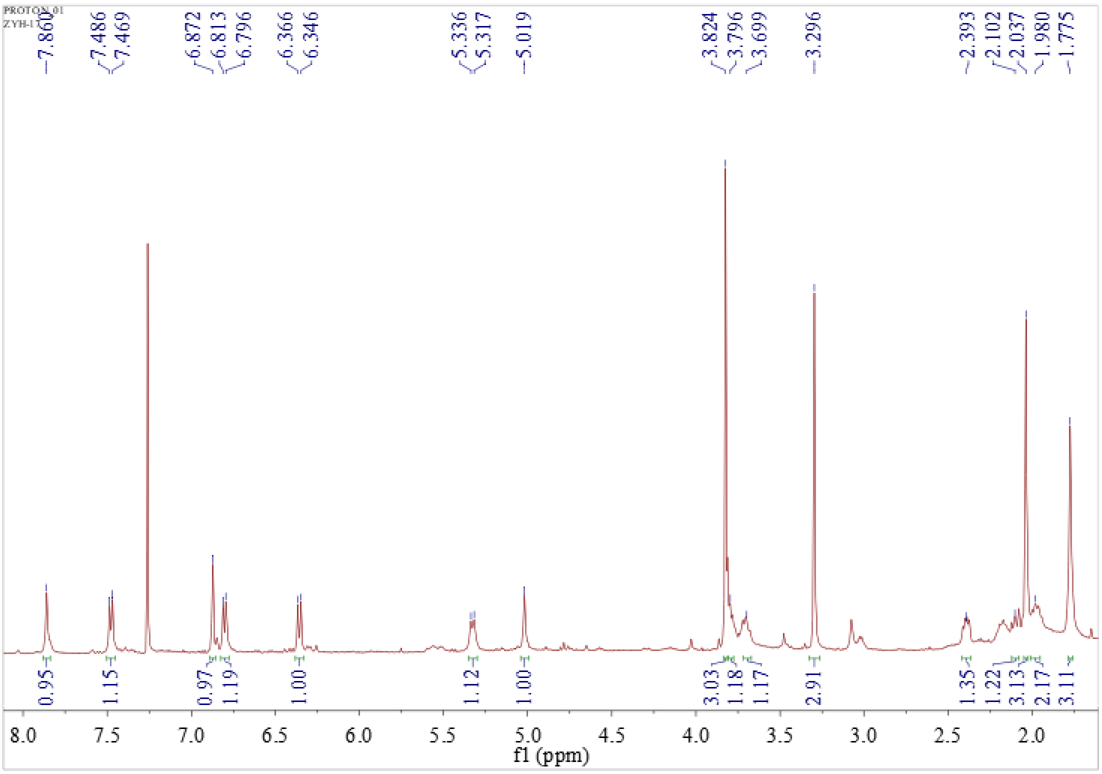

Figure S10. ^{1}H NMR (500 MHz, $CDCl_3$) spectrum of compound **2**.

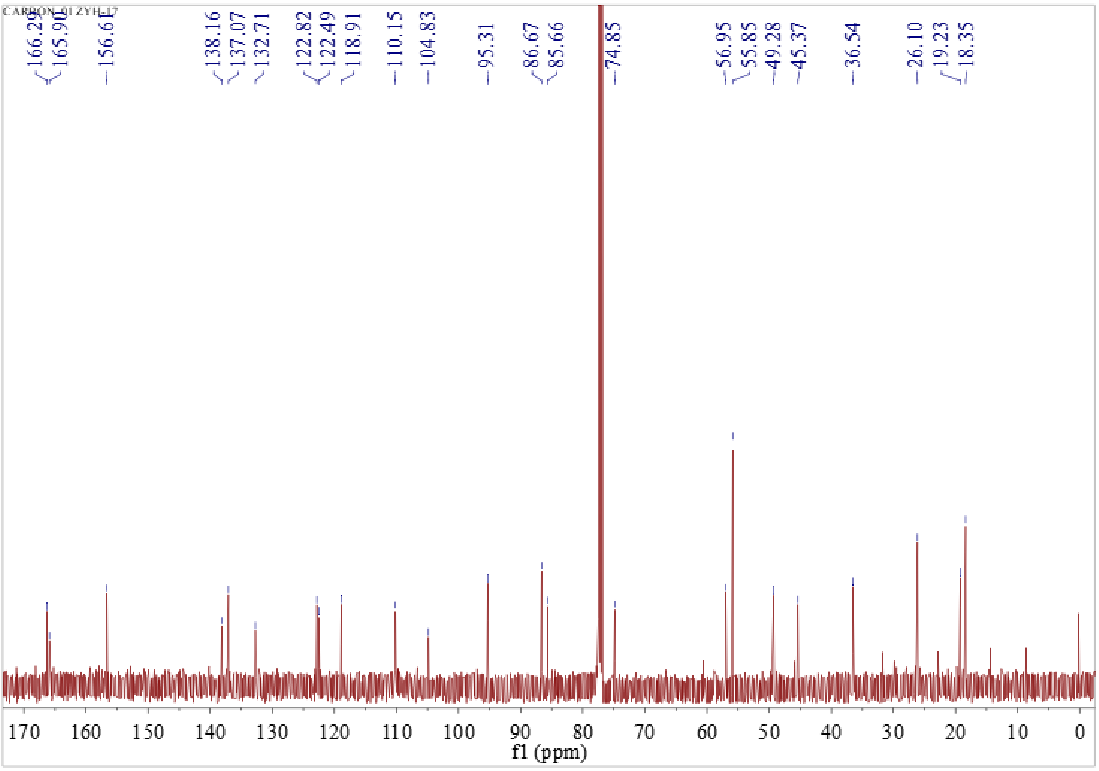

Figure S11. ^{13}C NMR (125 MHz, $CDCl_3$) spectrum of compound **2**.

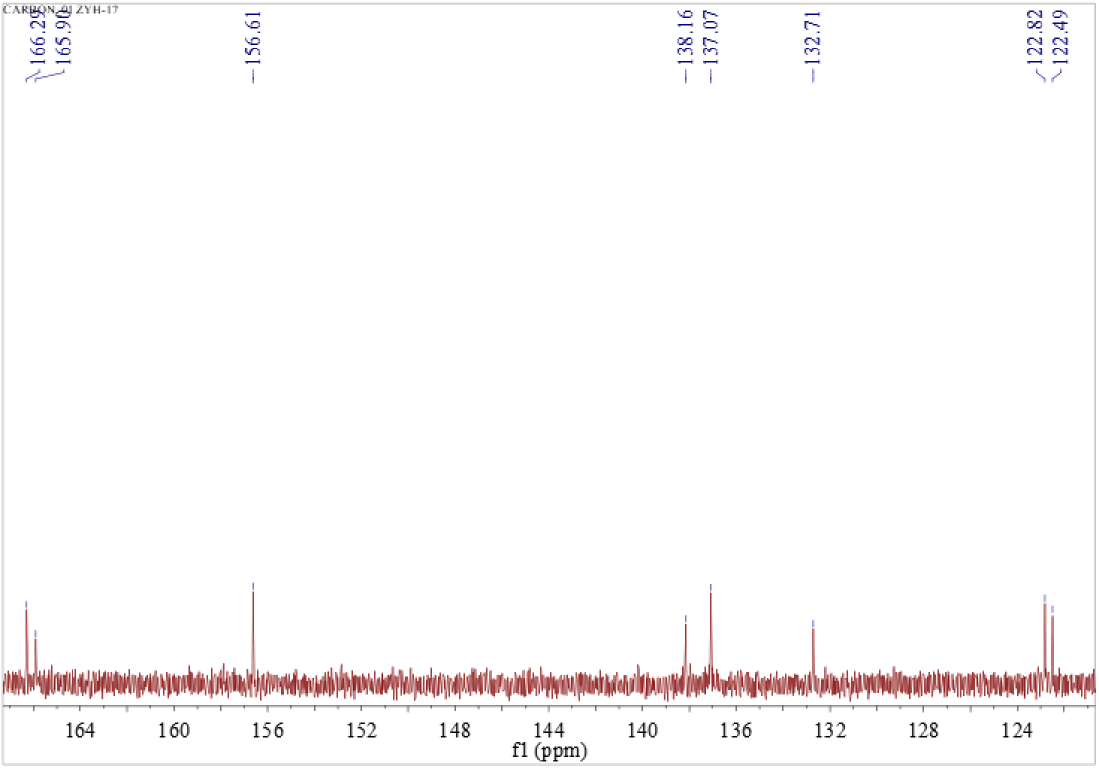

Figure S12. Partial ^{13}C NMR (125 MHz, $CDCl_3$) spectrum of compound **2**.

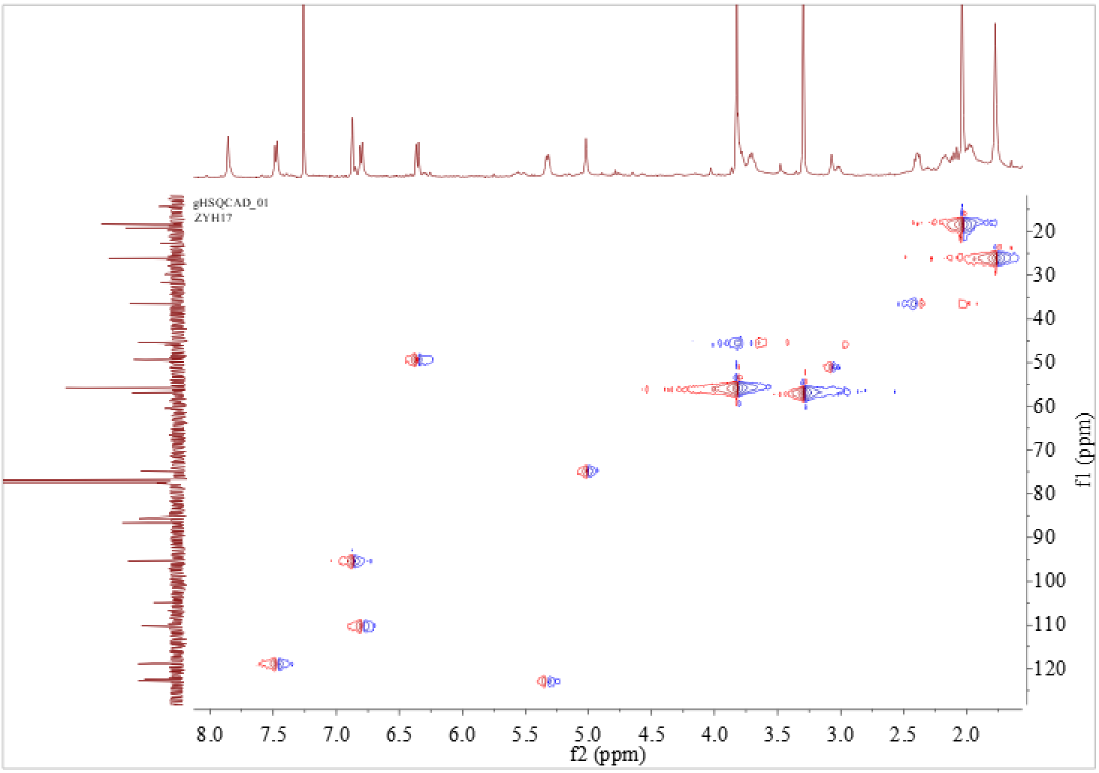

Figure S13. HSQC ($CDCl_3$) spectrum of compound **2**.

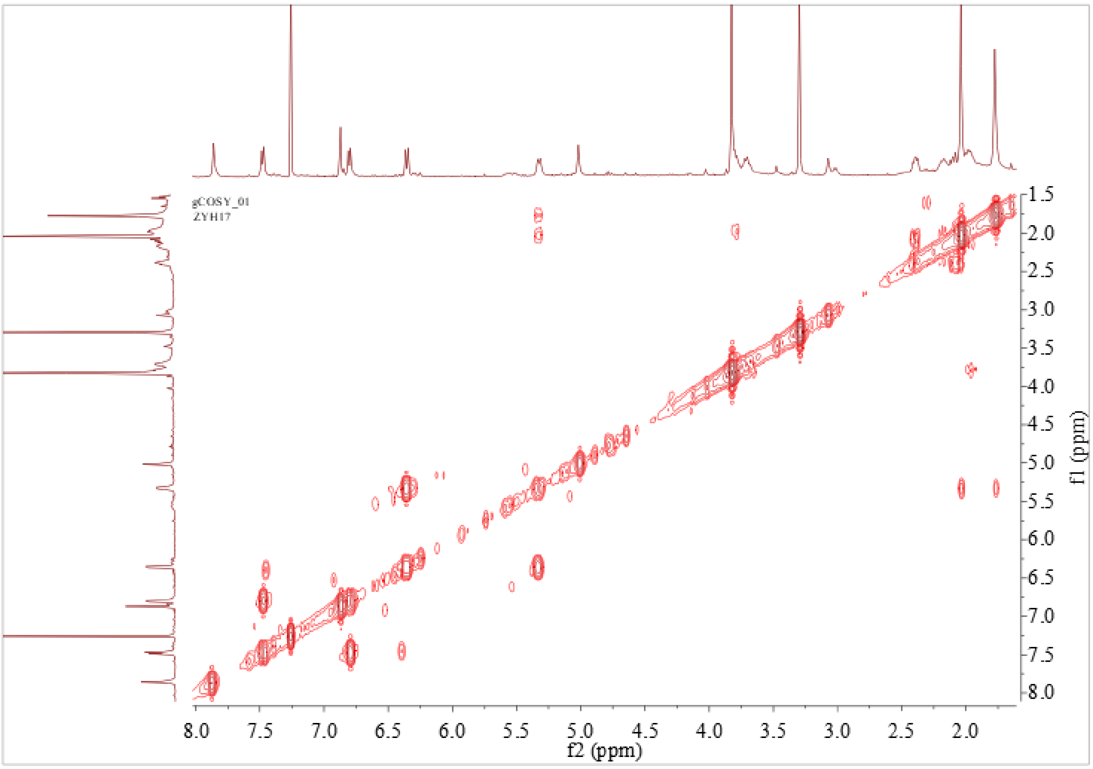

Figure S14. COSY ($CDCl_3$) spectrum of compound **2**.

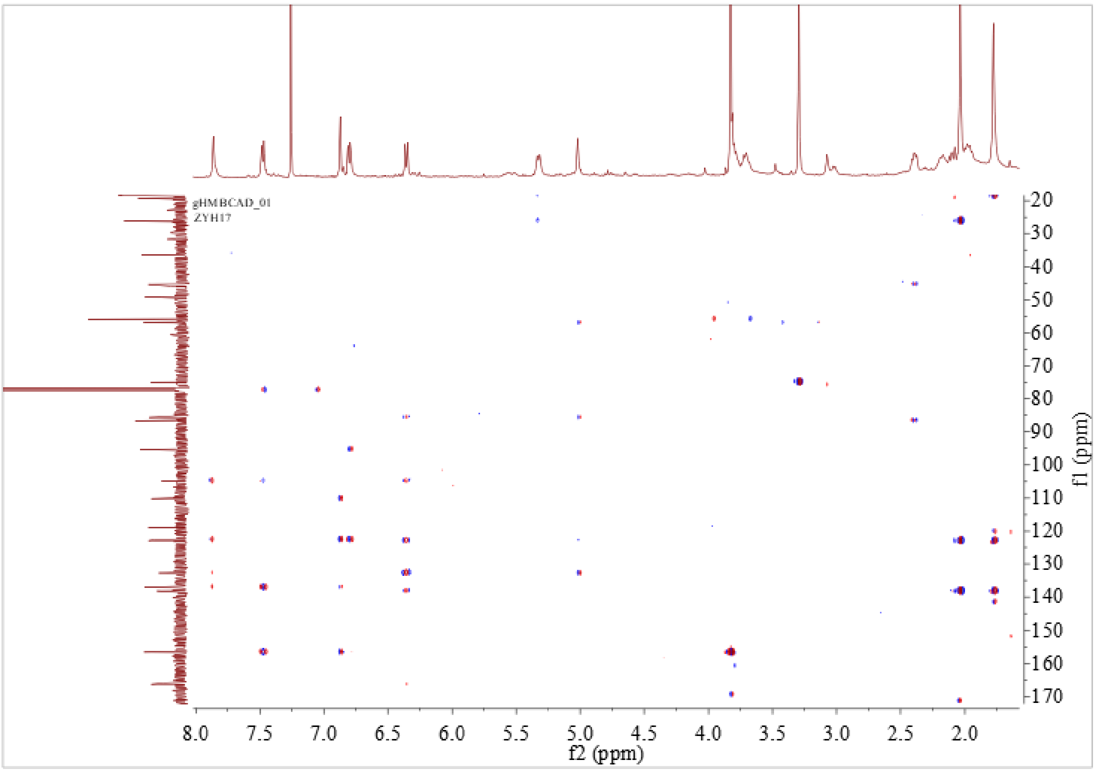

Figure S15. HMBC ($CDCl_3$) spectrum of compound **2**.

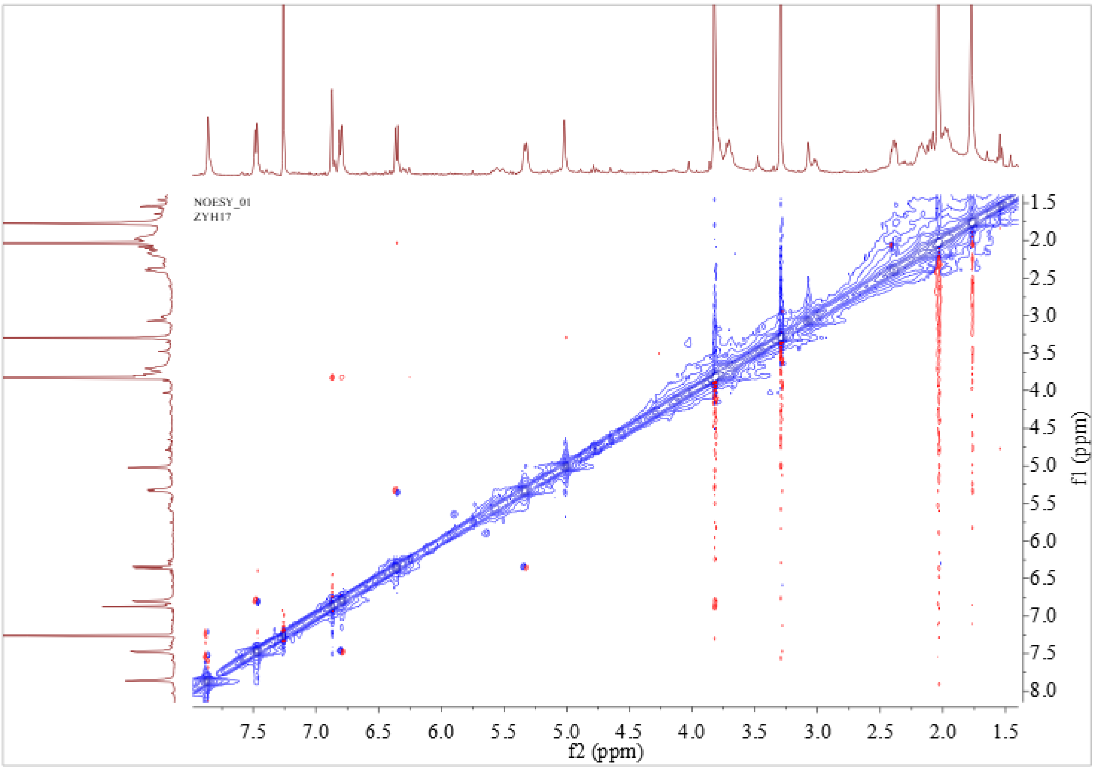

Figure S16. NOESY ($CDCl_3$) spectrum of compound **2**.

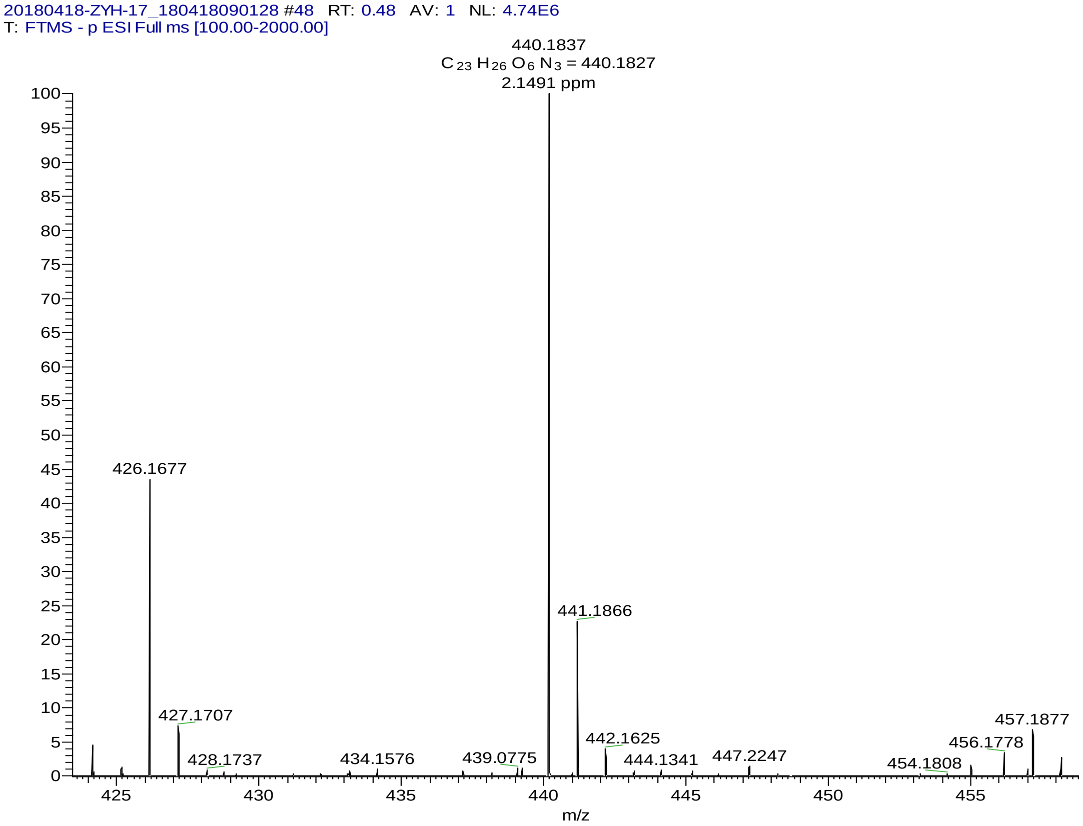

Figure S17. HRESIMS spectrum of compound **2**.

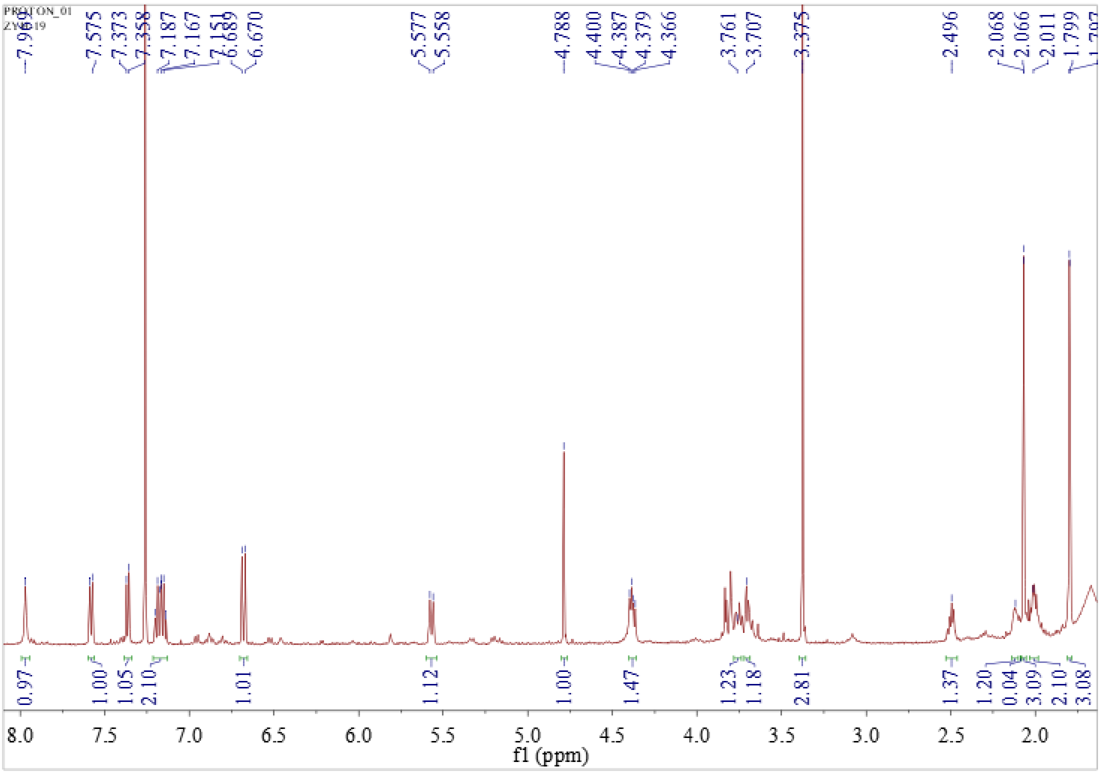

Figure S18. ^{1}H NMR (500 MHz, $CDCl_3$) spectrum of compound **3**

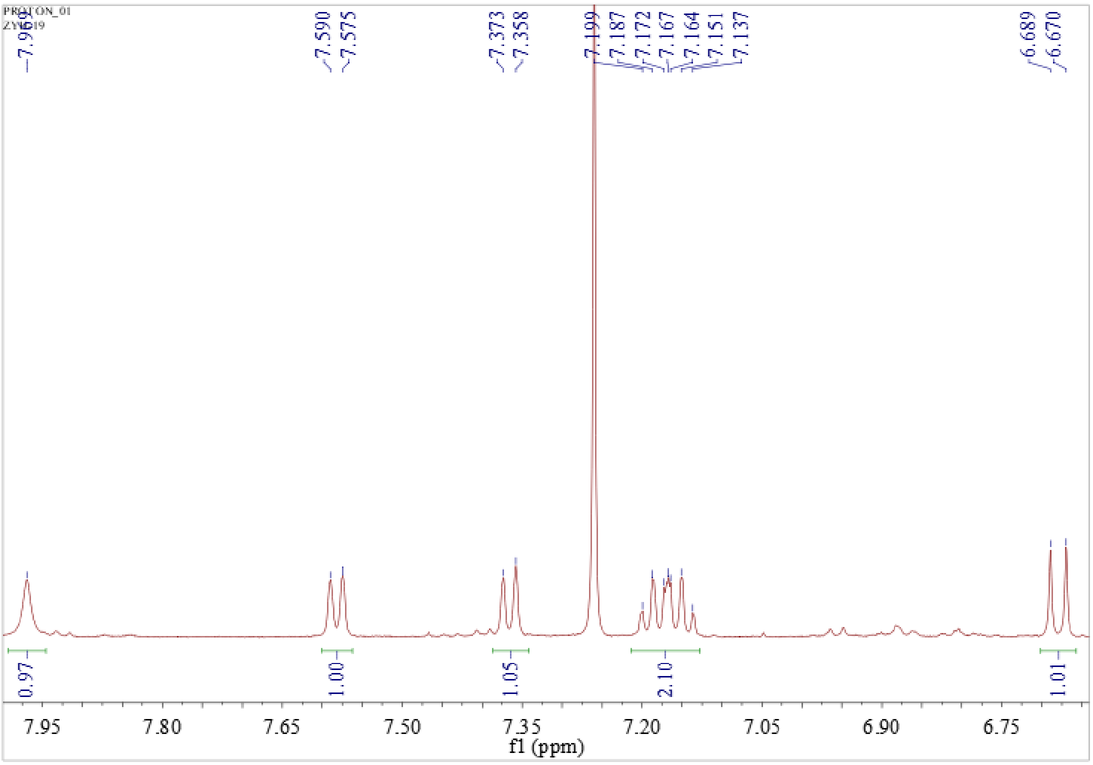

Figure S19. Partial ^{1}H NMR (500 MHz, $CDCl_3$) spectrum of compound **3**.

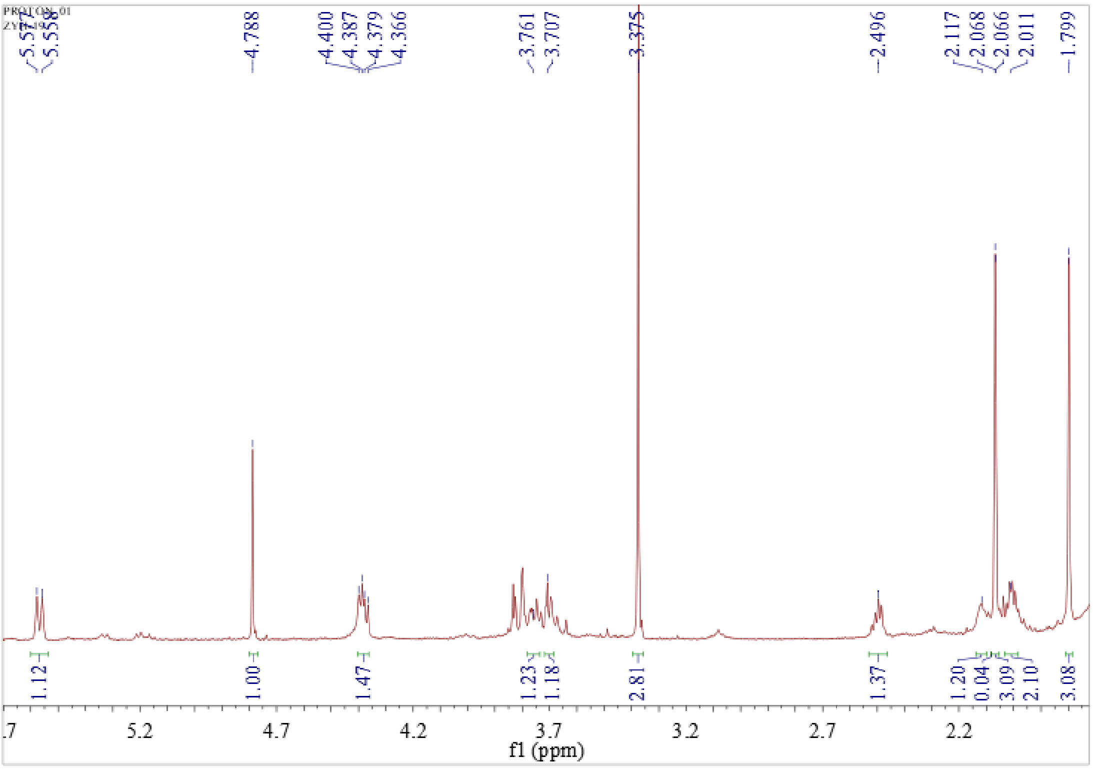

Figure S20. Partial ^{1}H NMR (500 MHz, $CDCl_3$) spectrum of compound **3**.

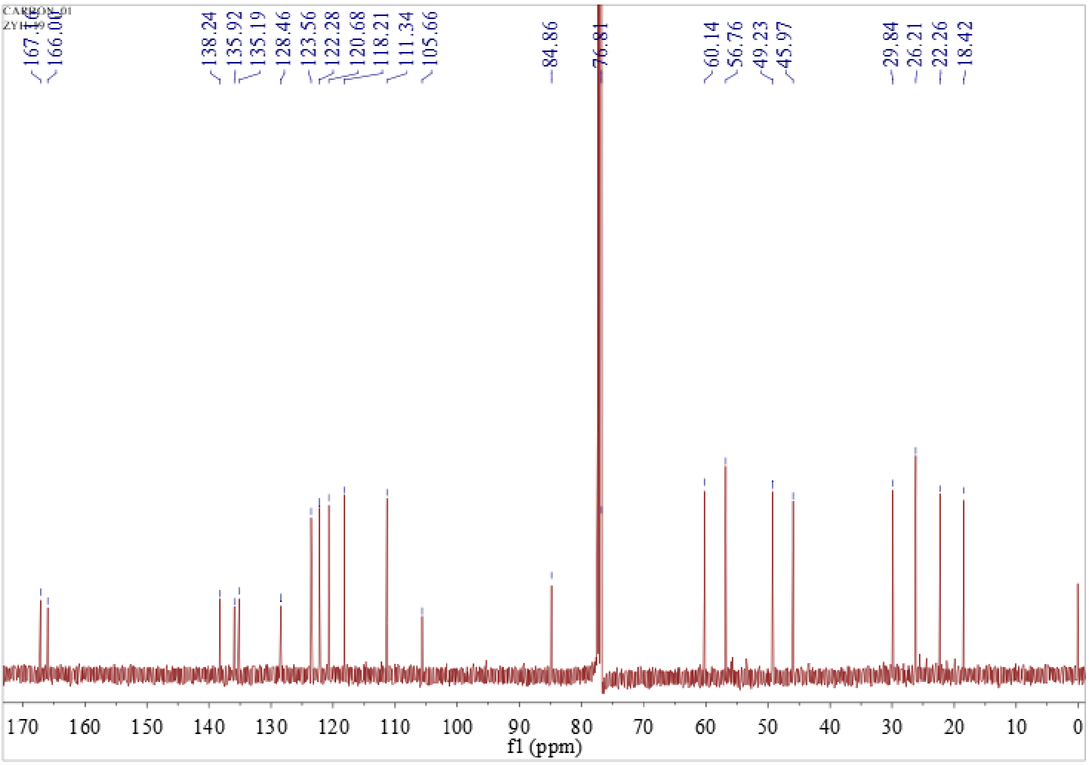

Figure S21. ^{13}C NMR (125 MHz, $CDCl_3$) spectrum of compound **3**

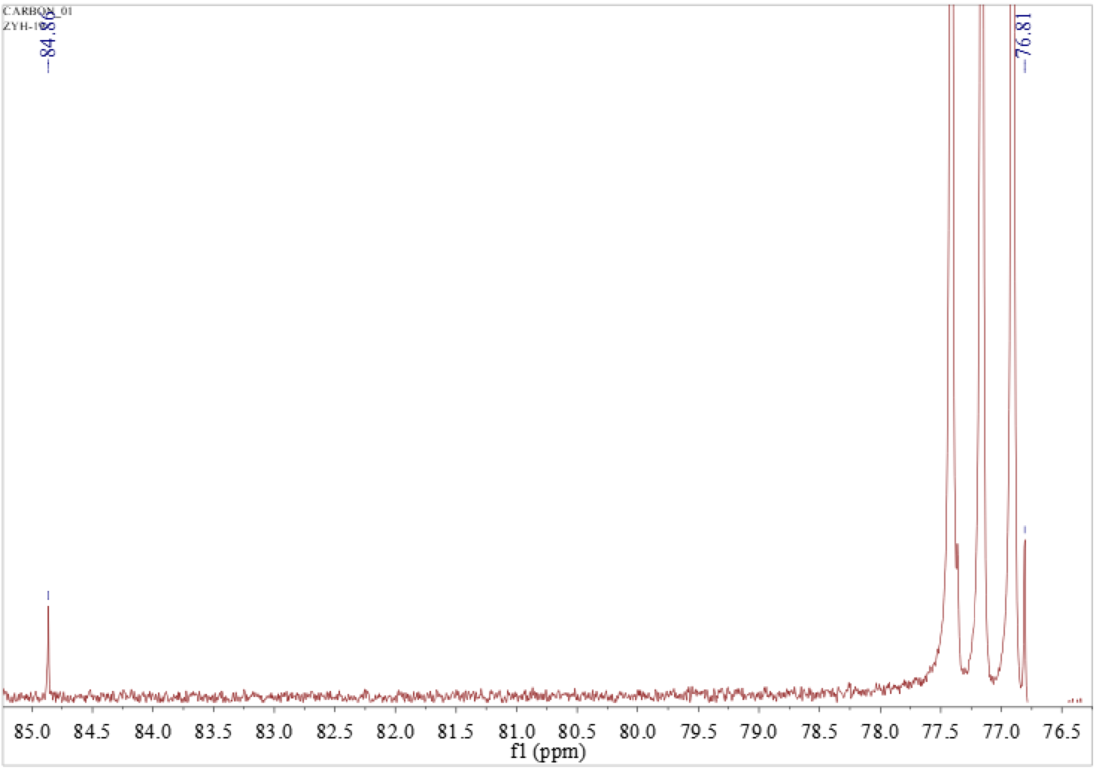

Figure S22. Partial ^{13}C NMR (125 MHz, $CDCl_3$) spectrum of compound **3**.

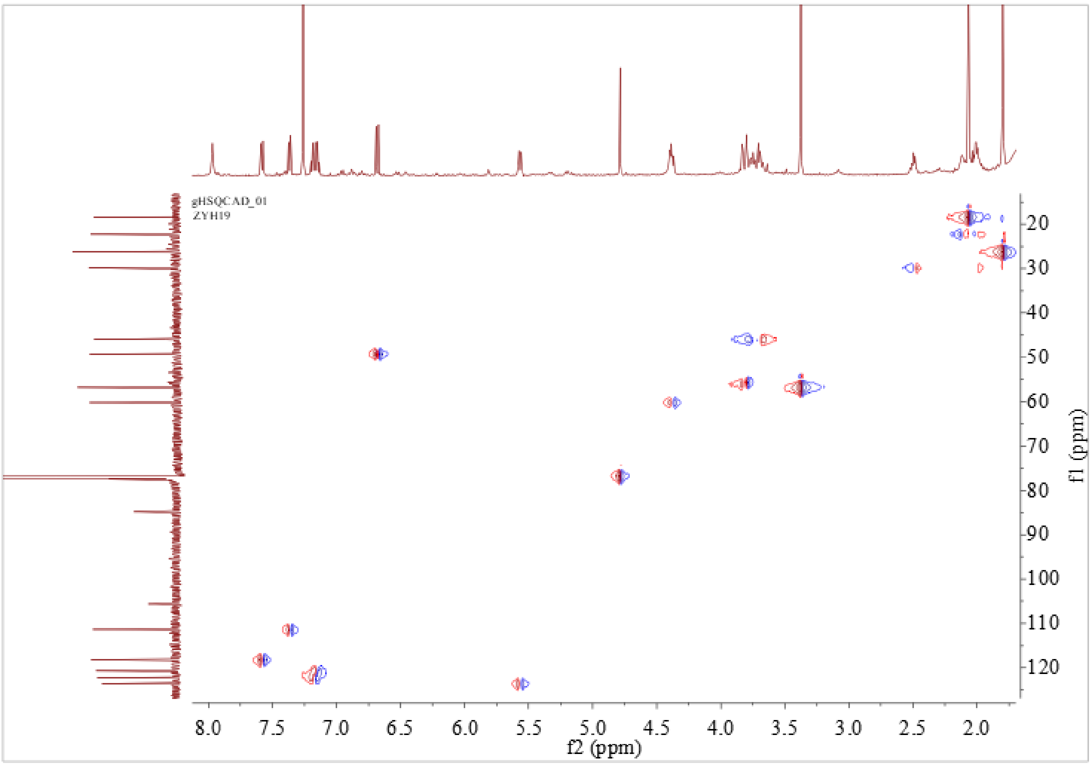

Figure S23. HSQC ($CDCl_3$) spectrum of compound **3**.

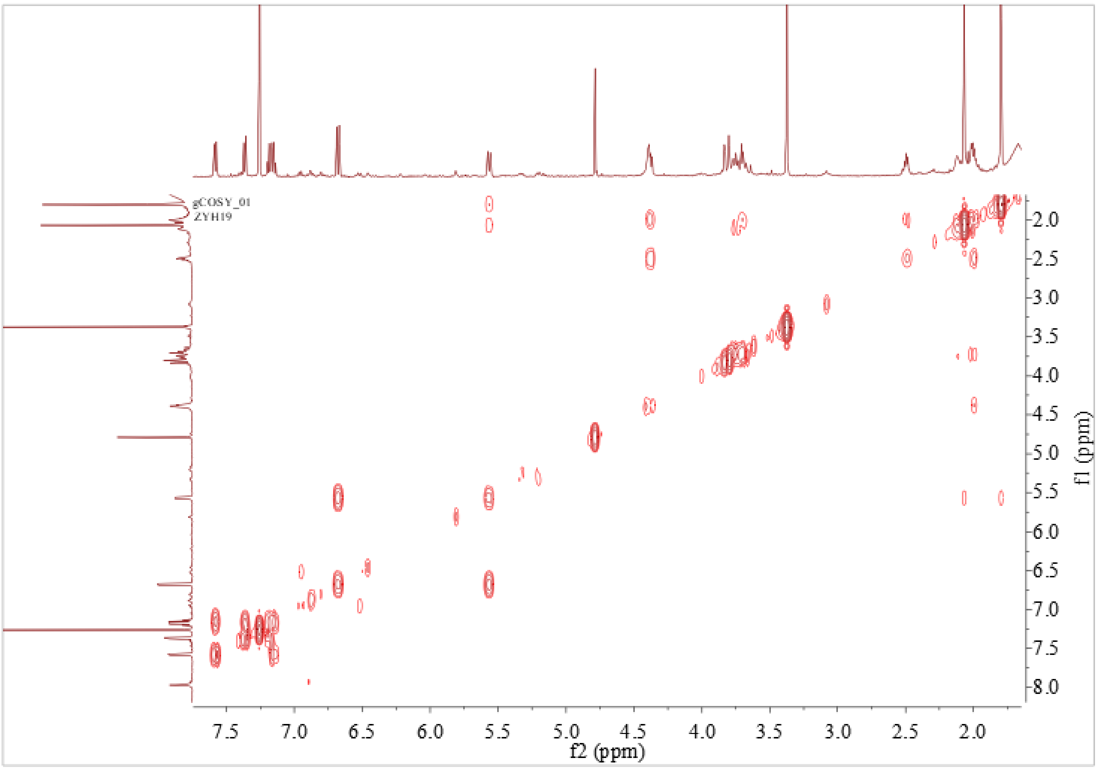

Figure S24. COSY ($CDCl_3$) spectrum of compound **3**.

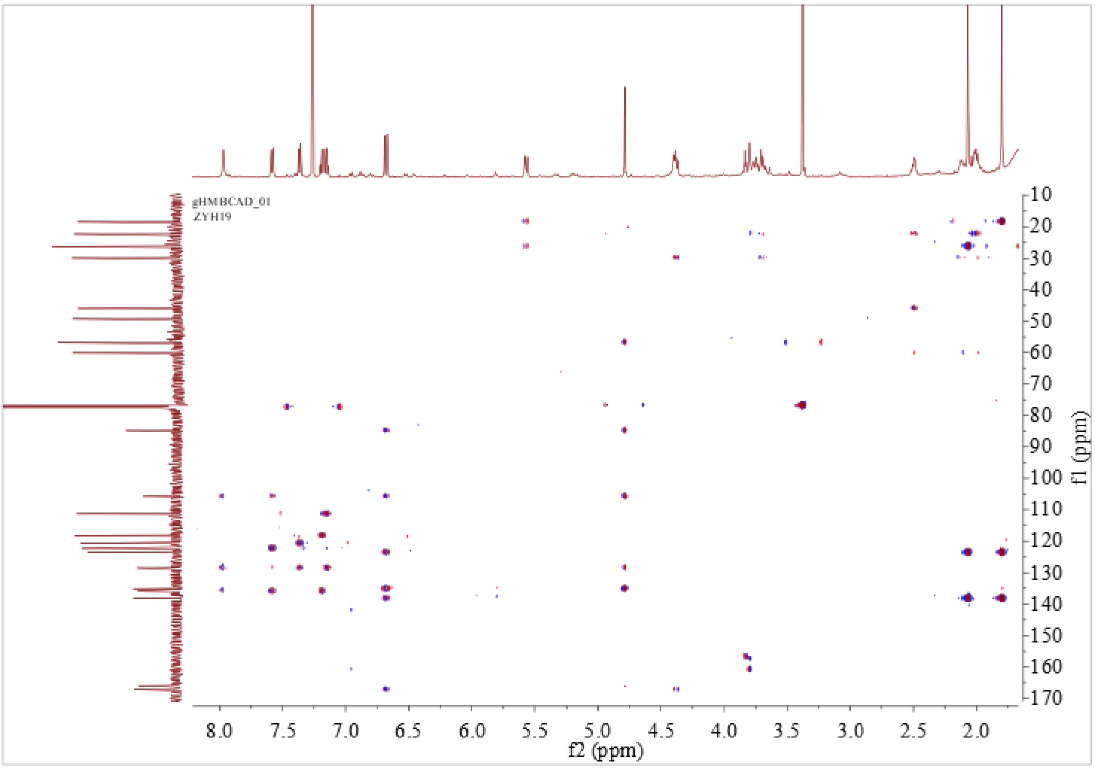

Figure S25. HMBC ($CDCl_3$) spectrum of compound **3**.

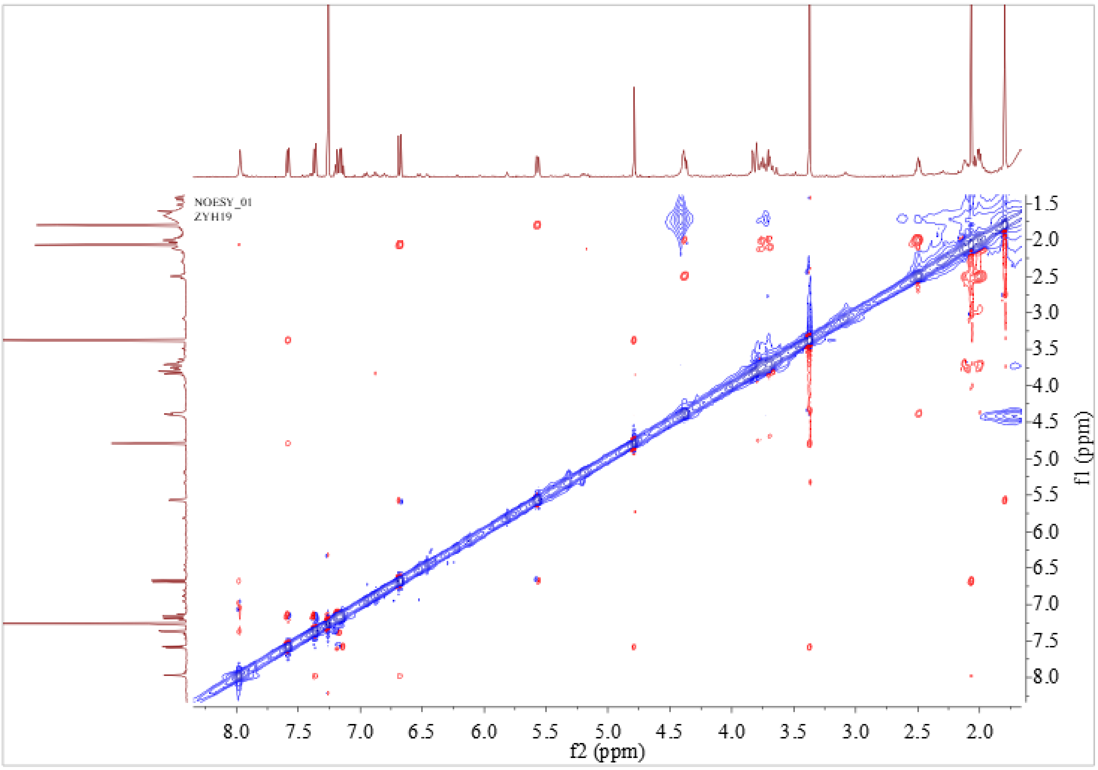

Figure S26. NOESY ($CDCl_3$) spectrum of compound **3**.

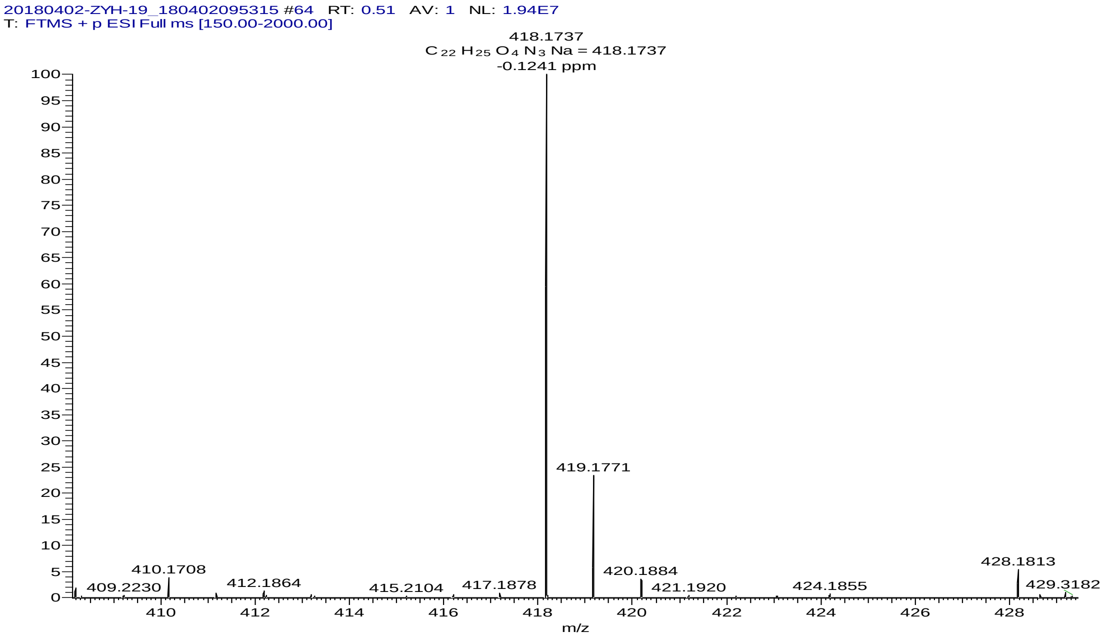

Figure S27. HRESIMS spectrum of compound **3**.

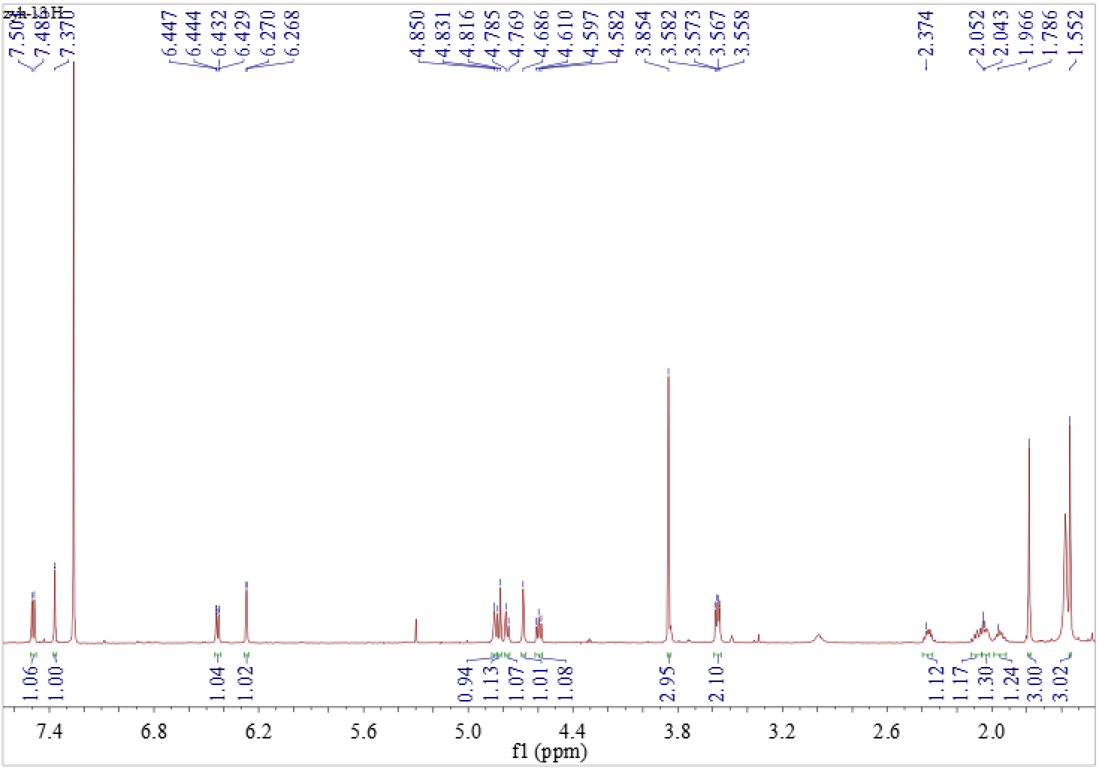

Figure S28. ^{1}H NMR (600 MHz, $CDCl_3$) spectrum of compound **4**.

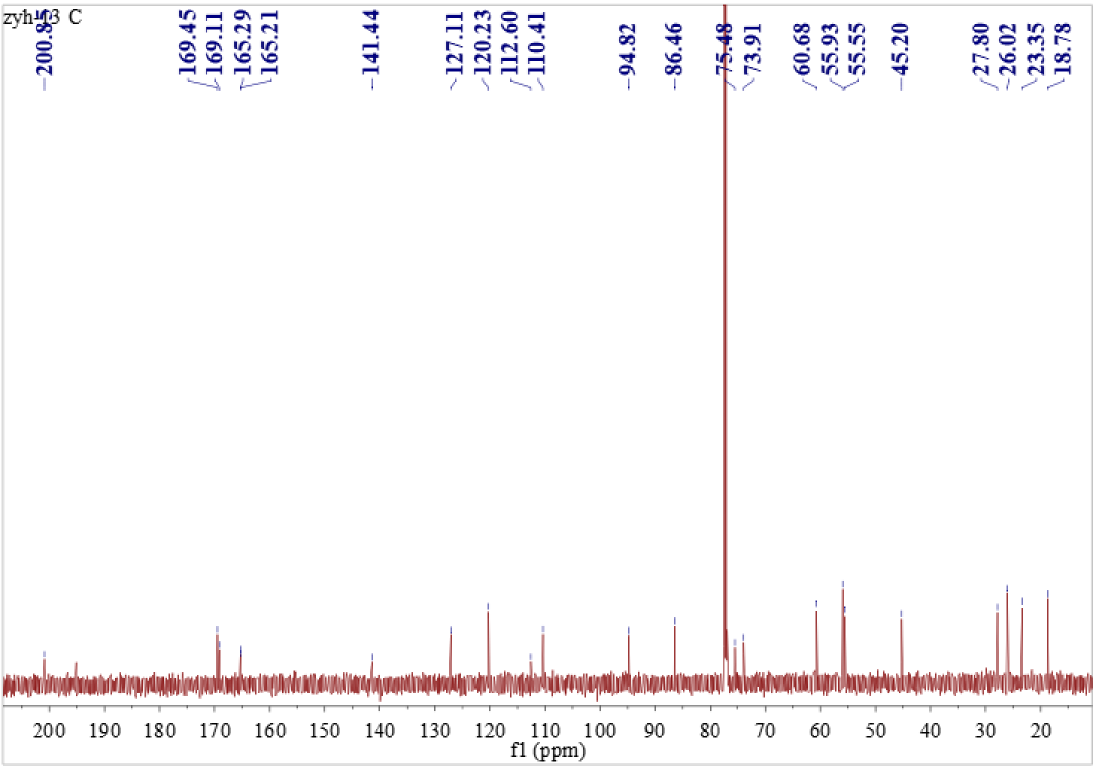

Figure S29. ^{13}C NMR (150 MHz, $CDCl_3$) spectrum of compound **4**.

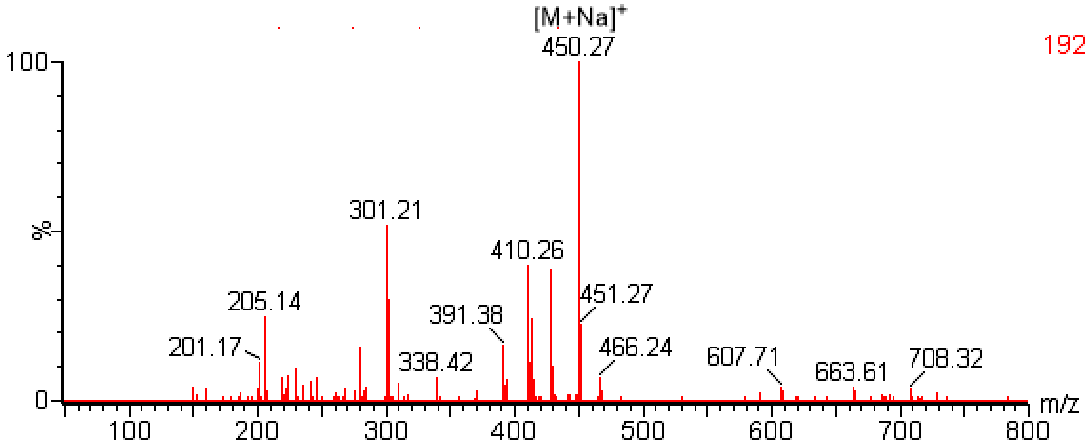

Figure S30. ESIMS spectrum of compound **4**.

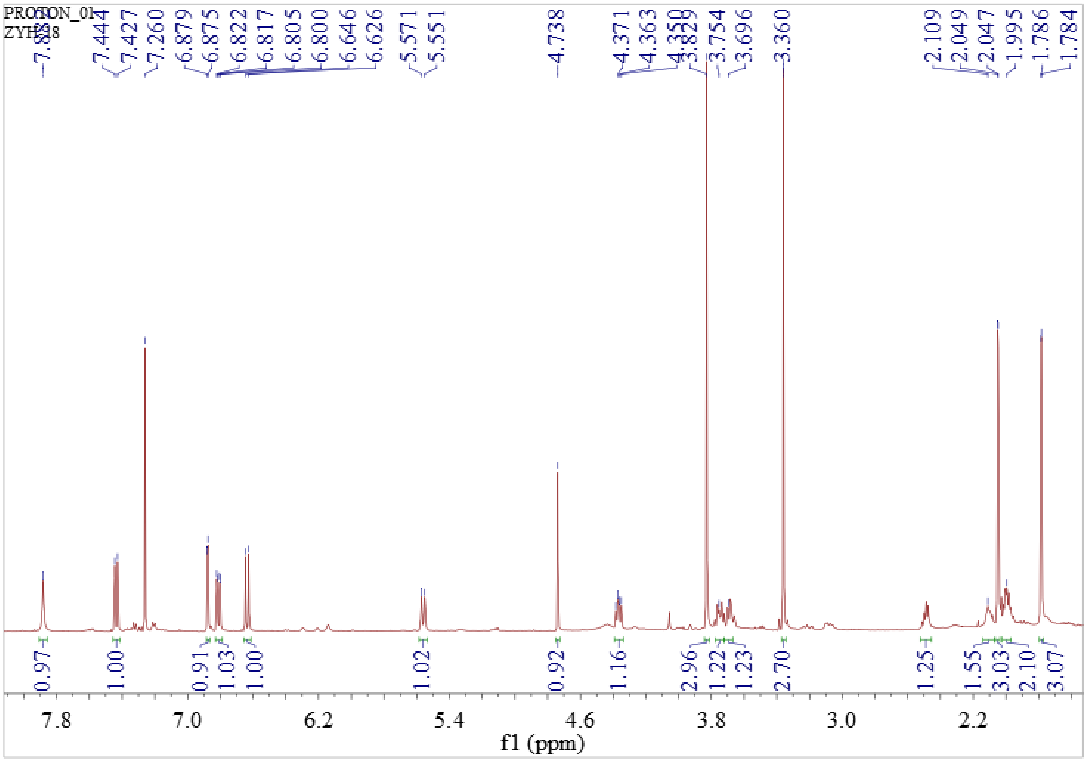

Figure S31. ^{1}H NMR (500 MHz, $CDCl_3$) spectrum of compound **5**.

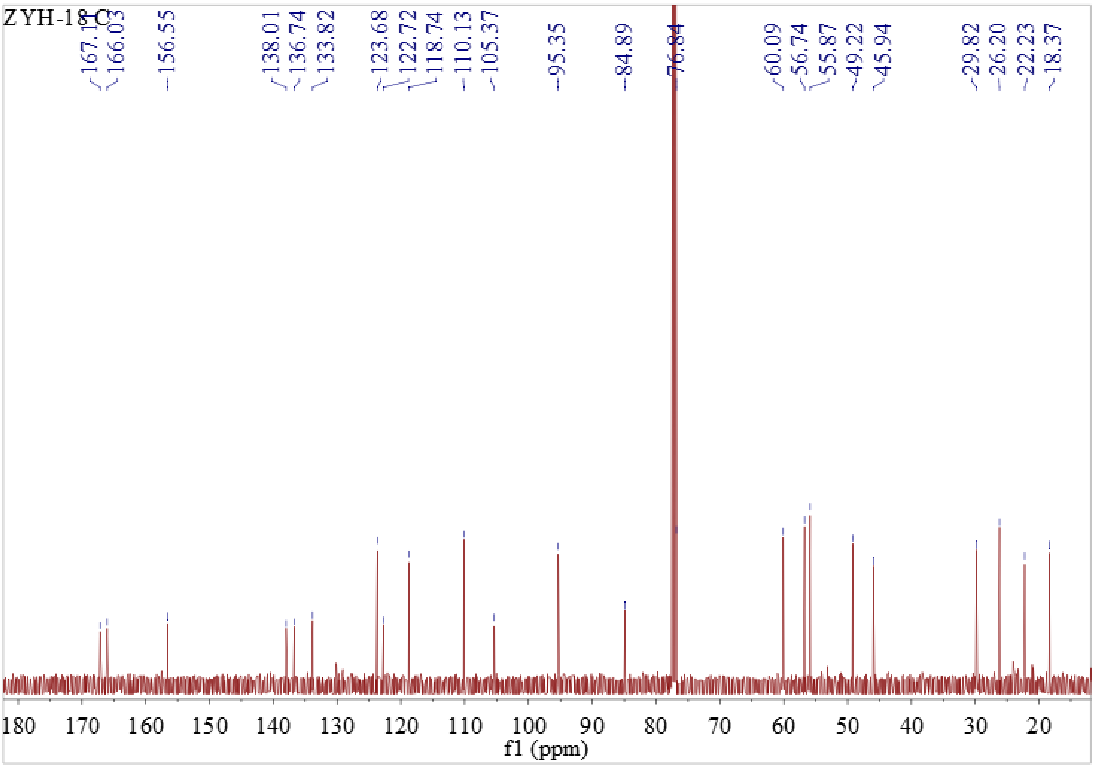

Figure S32. ^{13}C NMR (125 MHz, $CDCl_3$) spectrum of compound **5**

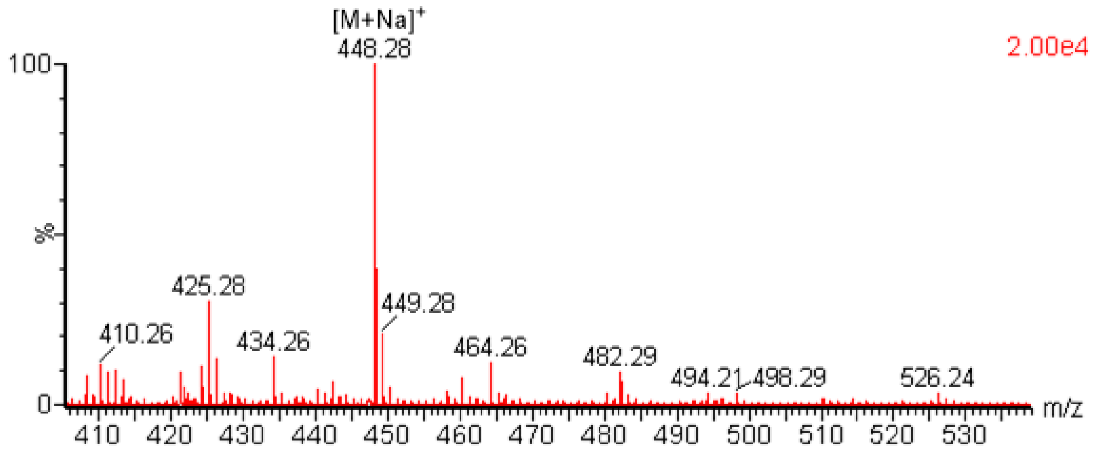

Figure S33. ESIMS spectrum of compound **5**.

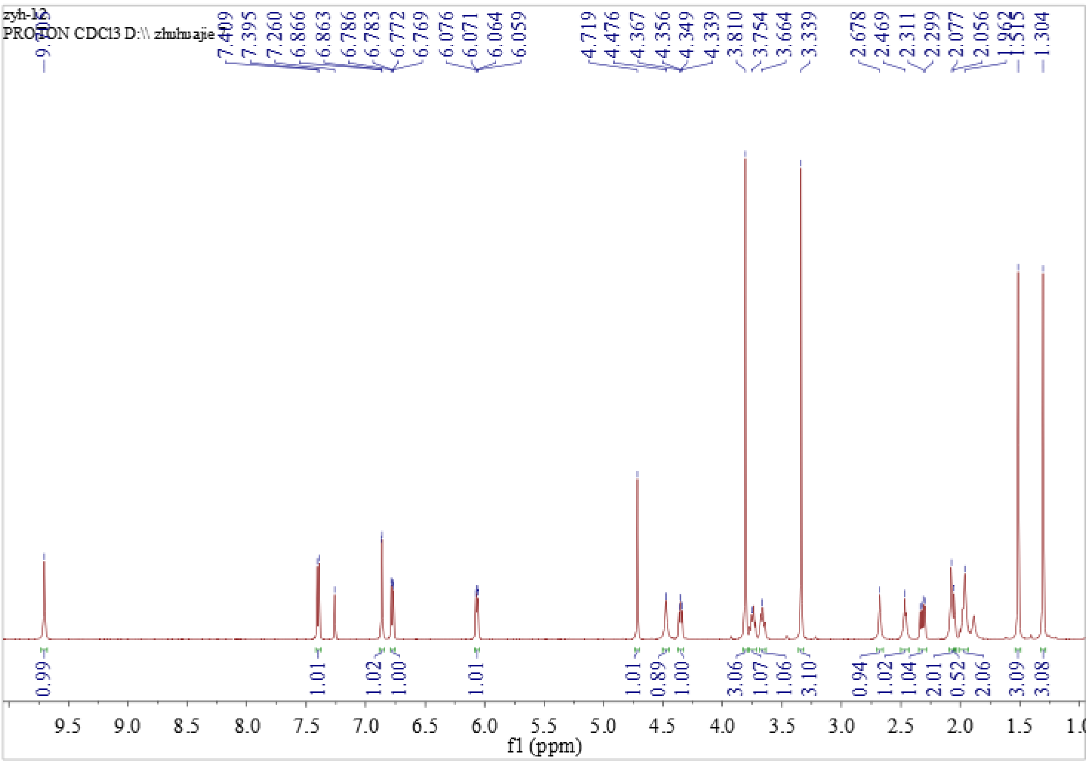

Figure S34. ^{1}H NMR (600 MHz, $CDCl_3$) spectrum of compound **6**.

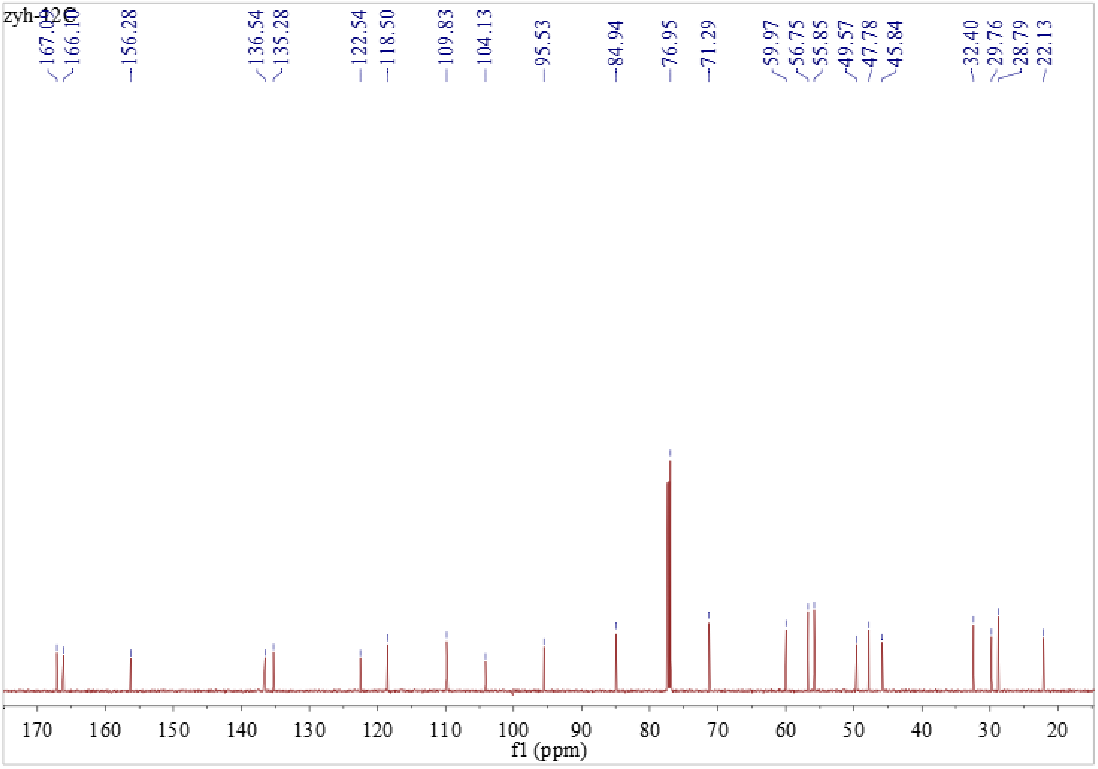

Figure S35. ^{13}C NMR (150 MHz, $CDCl_3$) spectrum of compound **6**.

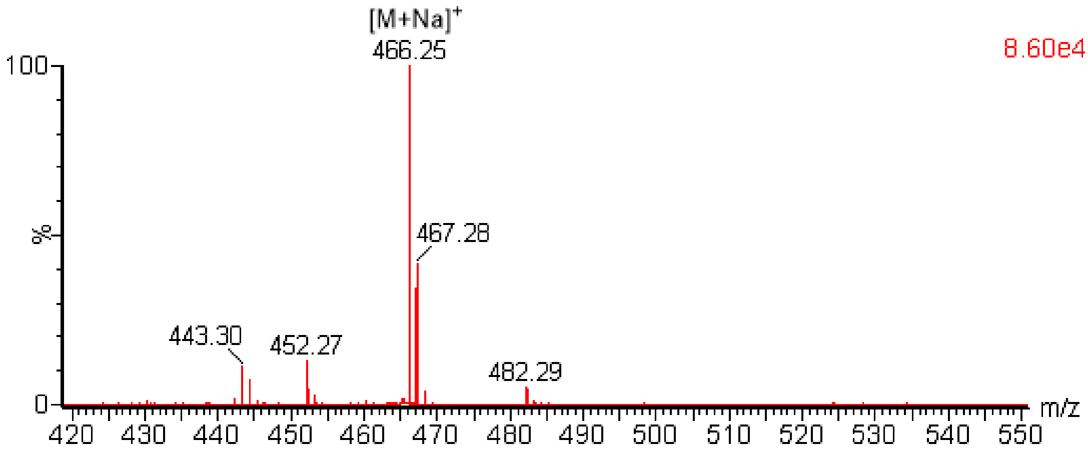

Figure S36. ESIMS spectrum of compound **6**.

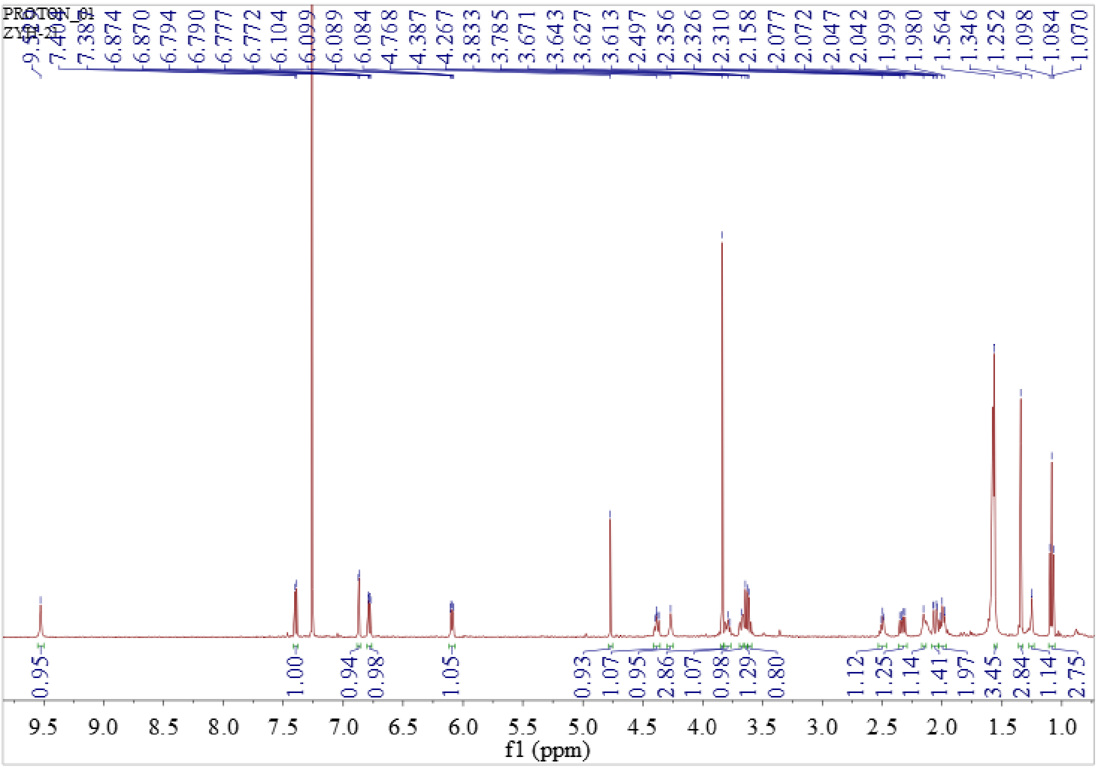

Figure S37. ^{1}H NMR (500 MHz, $CDCl_3$) spectrum of compound **7**.

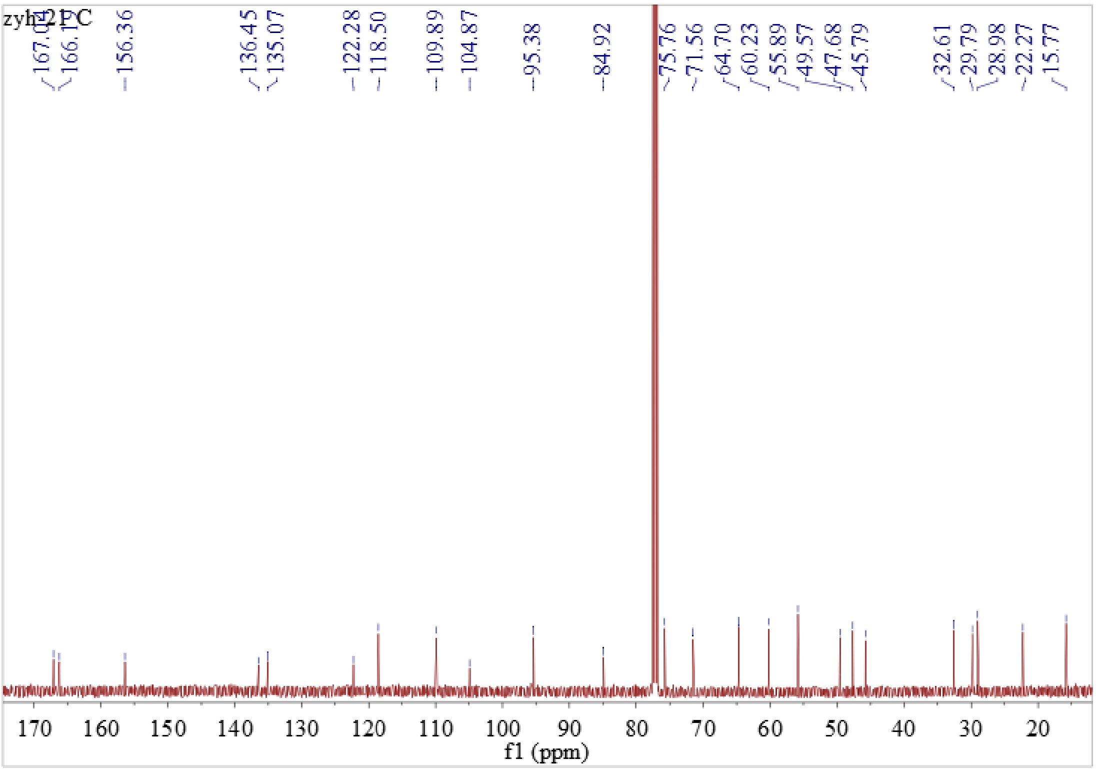

Figure S38. ^{13}C NMR (125 MHz, $CDCl_3$) spectrum of compound **7**.

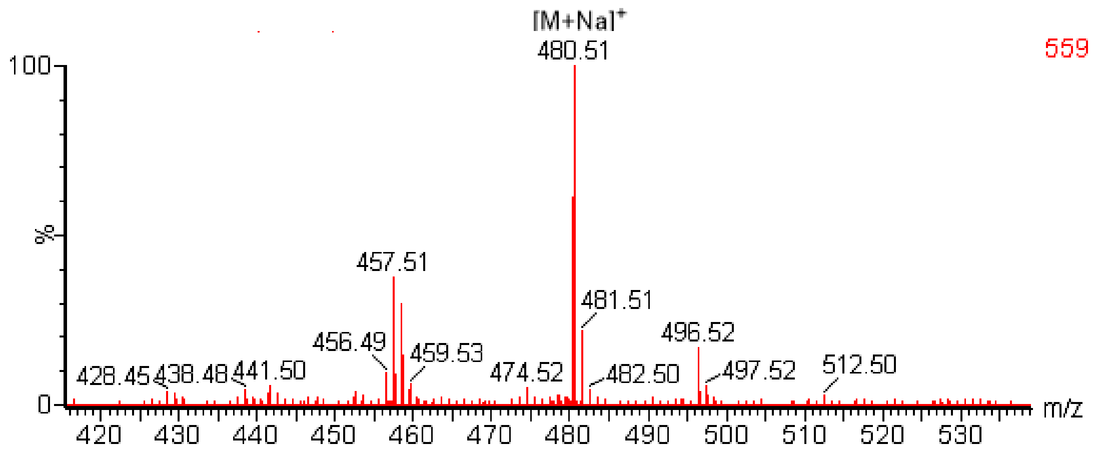

Figure S39. ESIMS spectrum of compound **7**.

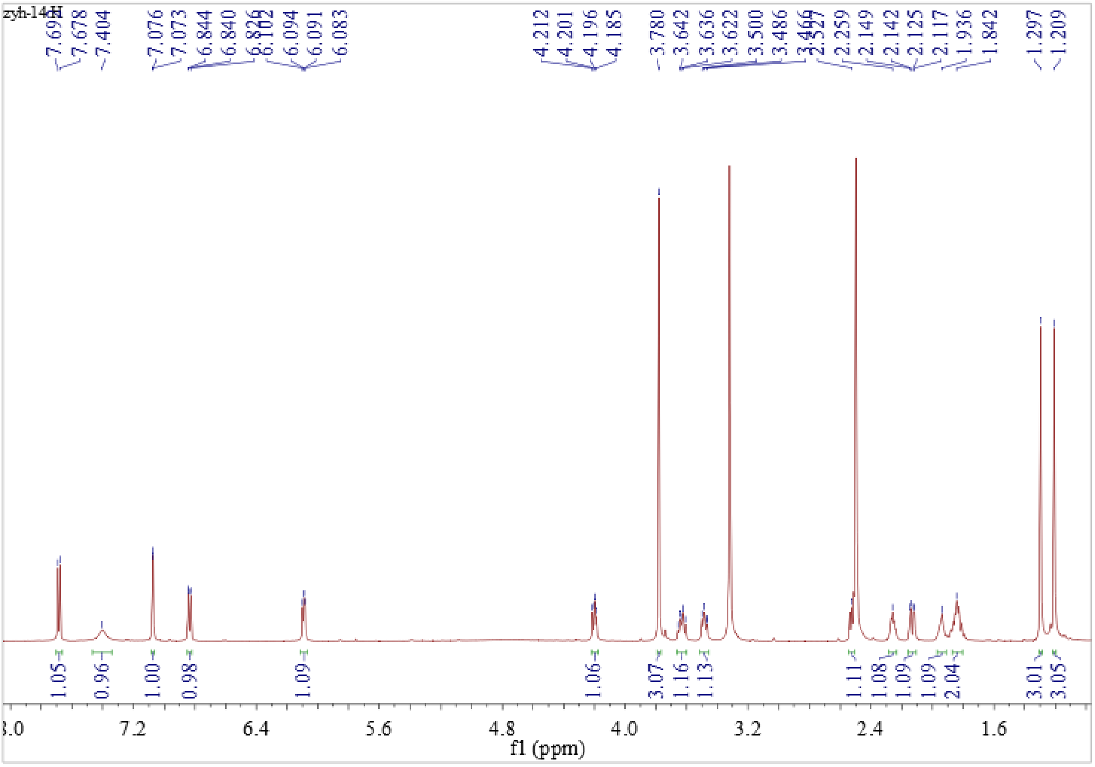

Figure S40. ^{1}H NMR (600 MHz, DMSO-d_6) spectrum of compound **8**.

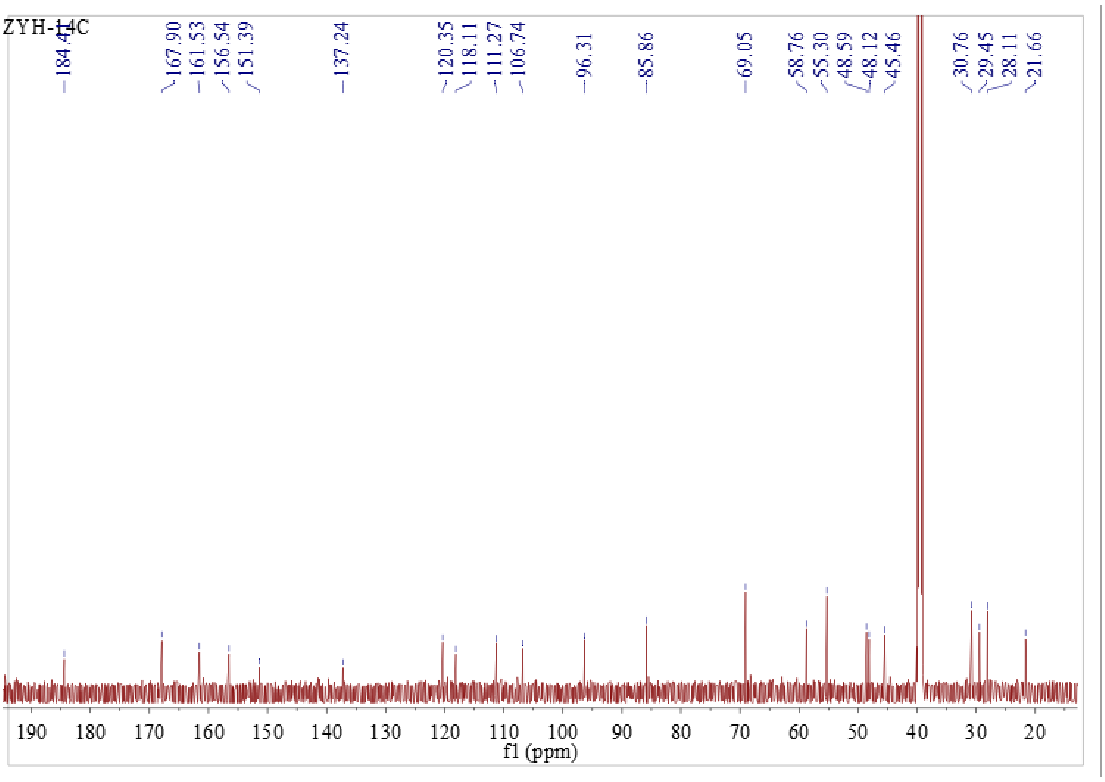

Figure S41. ^{13}C NMR (150 MHz, DMSO-d_6) spectrum of compound **8**.

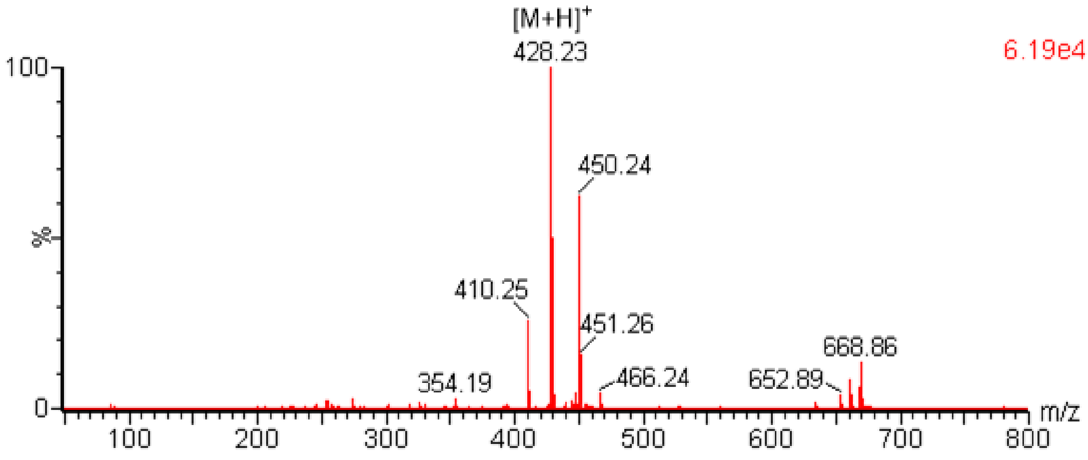

Figure S42. ESIMS spectrum of compound **8**.

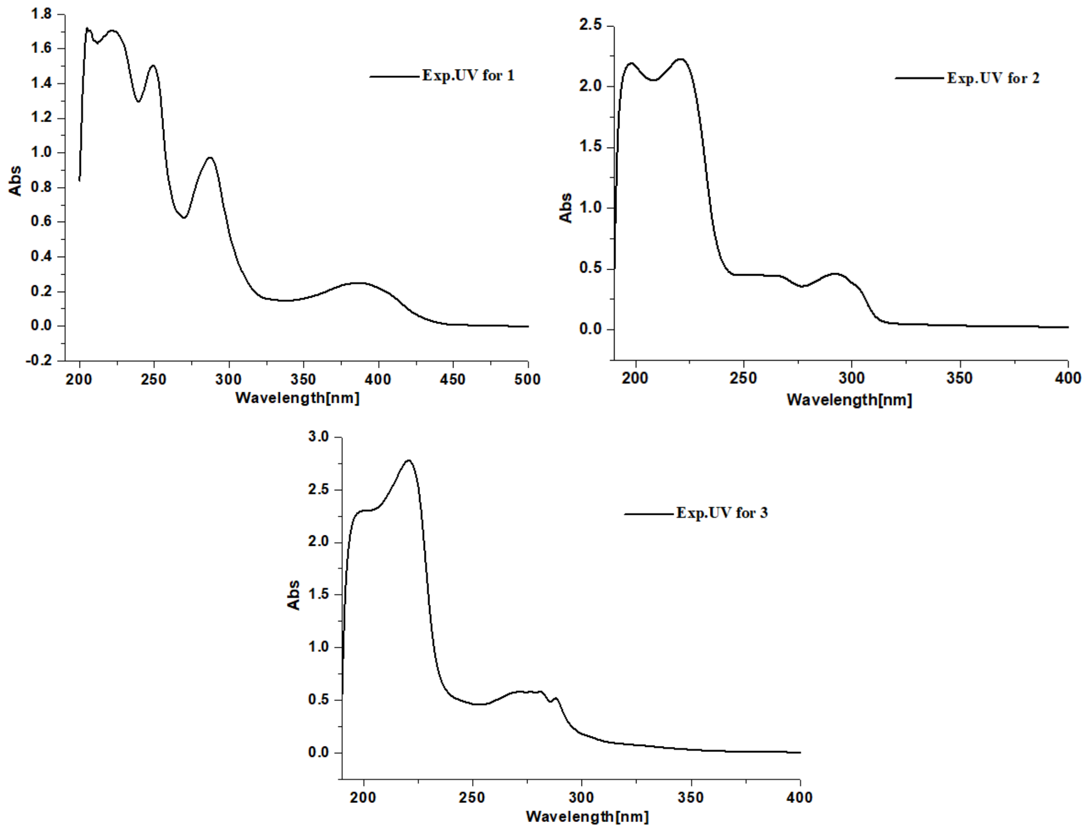

Figure S43. UV spectra of compounds **1**–**3**.

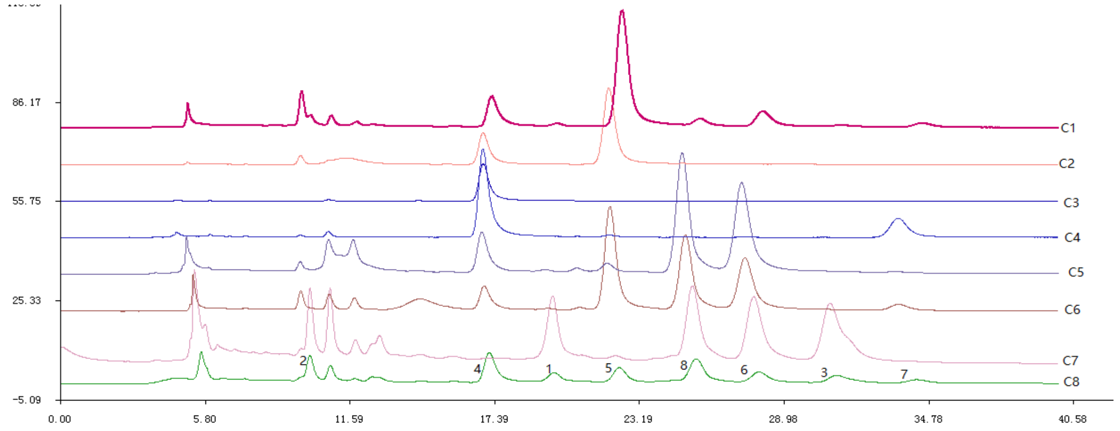

Figure S44. HPLC at 254 nm of the fermentation extracts from cultures in different media (C1-C8 t_R 0-5 min: 20% MeOH in H_2O, t_R 5-30 min: 20%-100% MeOH in H_2O, t_R 30-45 min:100 % MeOH, v = 2 mL/min).

C1: 80 mL water, 80 g rice, and 2.6 g $MgCl_2$ in 1 L Erlenmeyer flasks.

C2: 80 mL water and 80 g rice in 1 L Erlenmeyer flasks.

C3: 300 mL PDB in 1 L Erlenmeyer flasks.

C4: 300 mL PDB and 10 g sea salt in 1 L Erlenmeyer flasks.

C5: 80 mL water, 80 g rice, and 2.6 g NaCl in 1 L Erlenmeyer flasks.

C6: 80 mL PYG and 80 g rice in 1 L Erlenmeyer flasks.

C7: 80 mL water, 80 g rice, $NaNO_3$ 0.3 g, KH_2PO_4 0.1 g, $MgSO_4{\cdot}7H_2O$ 0.5 g, NaCl 0.05 g, $FeSO_4$ 0.01 g, sucrose 3.0 g in 1 L Erlenmeyer flasks.

C8: 80 mL water, 80 g rice, and 0.8 g $MgCl_2$ in 1 L Erlenmeyer flasks.

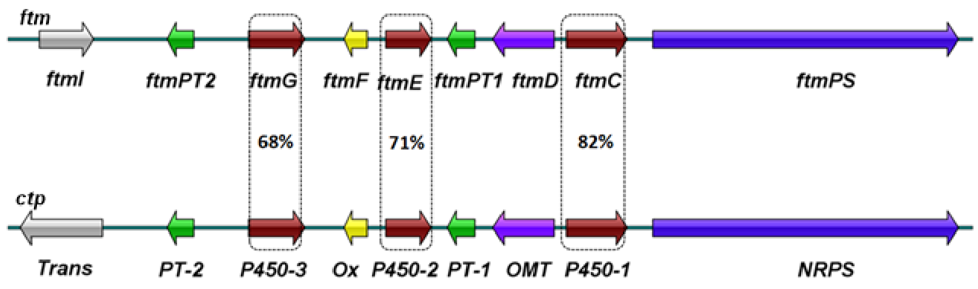

Figure S45. Comparison of our compounds BGC with fumitremorgin BGC

Table S1. Proposed NRPS biosynthetic gene clusters (NRPS-BGCs) predicted by fungiSMASH.

Gene cluster number	Scaffold location	Gene cluster type
cluster4	c00083_11315_2 47752..98270	Indole-t1PKS
cluster6	c00085_11318_8 22303..79507	NRPS
cluster13	c00112_11377_4 722..42612	NRPS
cluster17	c00135_11454_1 346838..402852	NRPS
cluster18	c00143_11485_2 1..29500	NRPS
cluster32	c00267_11710_1 259086..306783	NRPS
cluster33	c00267_11710_1 613477..672287	NRPS
cluster35	c00268_11712_3 35313..81905	NRPS
**cluster42**	**c00304_11766_5 1..41450**	**Indole-NRPS**
cluster45	c00311_11775_9 235173..278264	NRPS
cluster46	c00311_11775_9 405829..461882	NRPS
cluster48	c00330_11797_1 1..63658	Indole-NRPS
cluster50	c00345_11815_5 461680..508014	NRPS

Table S2. Proposed functions of genes in *ctp* gene clusters.

Gene	Protein size (aa)	Protein homolog	Identity/Coverage (%)	Function	Accession no.
ctpNRPS	2234	FtmA	67/99	Nonribosomal peptide synthetase	BAH23995.1
ctpP450-1	316	FtmC	82/99	Cytochrome P450	BAH23996.1
ctpOMT	410	FtmD	81/83	O-methyltransferase	BAH23997.1
ctpPT-1	462	FtmB	77/100	prenyltransferase	BAH23998.1
ctpP450-2	533	FtmE	71/98	Cytochrome P450	BAH23999.1
ctpOx	288	FtmF	77/99	Alpha-ketoglutarate dioxygenase	BAH24000.1
ctpP450-3	503	FtmG	68/98	Cytochrome P450	BAH24001.1
ctpPT-2	393	FtmH	67/100	prenyltransferase	BAH24002.1
ctpPT-MFS	581	--	100/100	putative MFS toxin efflux pump	OOQ91430.1

Table S3. The coordinate for the lowest-energy conformer [(2*S*,8*S*,9*R*,12*R*,18*S*)-**1**] in ECD calculation.

	Coordinates (Angstroms) X	Y	Z
C	5.24025400	-0.60470700	-0.71747400
C	5.46623100	0.23255200	0.39856600
C	4.39484400	0.61339000	1.19870500
C	3.11072300	0.16928000	0.88323300
C	2.90216700	-0.68341900	-0.21855900
C	3.95447100	-1.07478700	-1.03439800
C	1.82286700	0.42835200	1.52043500
C	0.75066800	-0.23740000	0.60879800
N	1.56089200	-1.08036300	-0.29206400
C	-0.14398000	0.78046600	-0.17342800
N	-1.36550500	-0.03644300	-0.40998700
C	-1.46462300	-1.22014700	0.40601900
C	-0.26193100	-1.12347800	1.38074900
C	-2.38821900	0.36463200	-1.23374300
C	-3.63358800	-0.56551400	-1.17720400
N	-3.86199600	-0.97745200	0.22106800
C	-2.85331900	-1.28641800	1.08088100
C	-4.94042100	0.14986300	-1.56757900
C	-5.70025200	0.38172400	-0.24775600
C	-5.25419200	-0.79143000	0.63720900
O	-2.99966300	-1.57921100	2.26006900
O	-2.31536900	1.34462600	-1.95787200
O	-3.44741000	-1.65901800	-2.05424500
O	1.57258500	1.02109700	2.55427200
C	-0.46043500	2.04561800	0.58051900
C	-0.40947000	3.29940100	0.10526800
C	-0.75444800	4.46184100	1.00521700
C	-0.02841400	3.68996400	-1.30063600
O	-1.28719800	-2.38379800	-0.42379000
O	0.25751800	-2.39957800	1.68375300
O	6.22025300	-1.02135600	-1.55689600
C	7.56084500	-0.60773700	-1.31668000
H	6.46400800	0.58259800	0.63185400
H	4.54370100	1.26239000	2.05682100
H	3.82576400	-1.72870700	-1.89064200
H	1.16524800	-1.28621800	-1.20352600
H	0.32113500	1.00971900	-1.13420700
H	-0.56533600	-0.63848900	2.31113800
H	-5.48865300	-0.53310800	-2.22312800
H	-4.72662900	1.06607000	-2.12045500
H	-5.39180400	1.32771400	0.21207700
H	-6.78479100	0.41402500	-0.38769100
H	-5.83823700	-1.69836700	0.43197500
H	-5.28744900	-0.59698200	1.71118100
H	-2.68889700	-2.17699700	-1.71780600
H	-0.75531100	1.89982900	1.61721100
H	-0.99668200	4.13780100	2.02156800
H	0.08232700	5.17140000	1.06402800
H	-1.61143000	5.02203800	0.60666400
H	0.08567700	2.84160400	-1.97632200
H	-0.80280800	4.33824300	-1.72976400
H	0.90478400	4.27021800	-1.29959600
H	-0.99040500	-3.08023300	0.19778700
H	1.00271500	-2.51775200	1.05809200
H	8.15595300	-1.06622800	-2.10773300
H	7.65948800	0.48323500	-1.37253300
H	7.91948800	-0.95835700	-0.34127800

Table S4. The coordinate for the lowest-energy conformer [(8*S*,9*S*,12*R*,18*S*)-**2**] in ECD calculation.

	Coordinates (Angstroms)		
	X	Y	Z
C	5.32903200	-0.90956900	-0.06328100
C	4.62278400	0.26108900	-0.32249500
C	3.22765700	0.19832700	-0.22858300
C	2.52575400	-0.98736700	0.10040900
C	3.27592500	-2.14744700	0.35952200
C	4.65519500	-2.10273900	0.27903800
N	2.28949100	1.19640400	-0.41709300
C	1.02848900	0.68141500	-0.20758300
C	1.12176900	-0.65113200	0.09662900
C	-0.21782400	1.51781600	-0.26444600
N	-1.37005700	0.61399100	0.00717000
C	-1.17879100	-0.46407100	0.97583300
C	-0.08751200	-1.44788600	0.46331400
C	-2.46535200	0.73610500	-0.80759600
C	-3.45398000	-0.44188800	-0.81346100
N	-3.51812900	-1.11610500	0.48429000
C	-2.49140800	-1.20846000	1.33427300
C	-4.91144800	-0.05574400	-1.05626900
C	-5.68545800	-1.24319500	-0.45787300
C	-4.85627000	-1.66635400	0.76947000
C	-0.15527700	2.71283000	0.66217300
C	-0.29565300	4.00087100	0.31923900
C	-0.17783600	5.07820900	1.36965400
C	-0.58924500	4.51509200	-1.06732600
O	-0.67728400	-2.18767600	-0.62768700
O	-0.72279100	0.11050500	2.18526600
O	-2.53143000	-1.79170900	2.41459600
O	-2.61967300	1.66621900	-1.58041900
O	-3.06569200	-1.33789900	-1.83983200
O	6.69279600	-1.01144600	-0.11271700
C	7.44554800	0.13817500	-0.45683700
C	0.11466000	-3.26230200	-1.11993900
H	5.11930500	1.18757300	-0.58291100
H	2.78731400	-3.07863200	0.62761300
H	5.25515700	-2.98369900	0.47692500
H	2.48977700	2.17387800	-0.55576100
H	-0.37366800	1.85894300	-1.28811800
H	0.12825700	-2.15710300	1.27156100
H	-5.09427300	0.10419600	-2.11701300
H	-5.13396300	0.87400900	-0.52664400
H	-6.70864300	-0.98047200	-0.18417200
H	-5.73153800	-2.05890600	-1.18121000
H	-4.80338200	-2.74761300	0.90676700
H	-5.22289000	-1.23191500	1.70340300
H	0.03102300	2.45928900	1.70105200
H	0.05899700	4.67024600	2.35394800
H	0.60027100	5.80395600	1.10461100
H	-1.11258200	5.64494600	1.45183500
H	-0.82334300	3.73364100	-1.78770800
H	-1.44976500	5.19219900	-1.03933300
H	0.25217900	5.10588200	-1.44996900
H	-1.06777000	-0.45468600	2.89801200
H	-2.19238800	-1.70259600	-1.60225500
H	8.48910500	-0.17155300	-0.44232400
H	7.19053000	0.49942400	-1.45945200
H	7.29947200	0.94603600	0.26898700
H	1.04559100	-2.90007900	-1.56424300
H	0.34346300	-3.97406200	-0.31797600
H	-0.47734200	-3.76754000	-1.88304400

Table S5. Antibacterial activities data of compounds 1–8

Compd. MIC (μM)									
	**1**	**2**	**3**	**4**	**5**	**6**	**7**	**8**	CIP
B. megaterium	>25	>25	>25	>25	>25	>25	>25	>25	0.625
B. subtilis	>25	>25	>25	>25	>25	>25	>25	>25	0.039
E. coli	>25	>25	>25	>25	>25	>25	>25	>25	0.156
B. anthraci	>25	>25	>25	>25	>25	>25	>25	>25	0.078
B. cereus	>25	>25	>25	>25	>25	>25	>25	>25	0.313
B. paratyphosum B	>25	>25	>25	>25	>25	>25	>25	>25	0.078
E. aerogenes	>25	>25	>25	>25	>25	>25	>25	>25	0.002
M. lysodeikticus	>25	>25	>25	>25	>25	>25	>25	>25	0.078
M. luteus	>25	>25	>25	>25	>25	>25	>25	>25	0.02
P. vulgaris	>25	>25	>25	>25	>25	>25	>25	>25	0.156
S. dysenteriae	>25	>25	>25	>25	>25	>25	>25	>25	0.039
P. aeruginosa	>25	>25	>25	>25	>25	>25	>25	>25	0.005
S. aureus	>25	>25	>25	>25	>25	>25	>25	>25	0.039
S. typhi	>25	>25	>25	>25	>25	>25	>25	>25	0.078
V. anguillarum	>25	>25	>25	>25	>25	>25	>25	>25	0.039
V. parahemolyticus	>25	>25	>25	>25	>25	>25	>25	>25	0.156

Table S6. Cytotoxic activity data of compounds 1–8

Compd.	IC_{50} (μM)		
	HL-60	HCT-116	MCF-7
**1**	6.0	>10	>10
**2**	>10	>10	7.6
**3**	>10	>10	10.8
**4**	7.9	>10	>10
**5**	>10	>10	5.1
**6**	>10	>10	>10
**7**	>10	>10	>10
**8**	>10	>10	>10
**DDP**	1.14	3.36	2.59
